# CD163^ΔSRCR5^ MARC-145 Cells Resist PRRSV-2 Infection via Inhibiting Virus Uncoating, Which Requires the Interaction of CD163 With Calpain 1

**DOI:** 10.3389/fmicb.2019.03115

**Published:** 2020-01-13

**Authors:** Piao Yu, Ruiping Wei, Wenjuan Dong, Zhenbang Zhu, Xiaoxiao Zhang, Yaosheng Chen, Xiaohong Liu, Chunhe Guo

**Affiliations:** State Key Laboratory of Biocontrol, School of Life Sciences, Sun Yat-sen University, Guangzhou, China

**Keywords:** PRRSV, CD163, SRCR5, calpain 1, resistance

## Abstract

Porcine alveolar macrophages without the CD163 SRCR5 domain are resistant to porcine reproductive and respiratory syndrome virus (PRRSV) infection. However, whether the deletion of CD163 SRCR5 in MARC-145 cells confers resistance to PRRSV and interaction of which of the host proteins with CD163 is involved in virus uncoating remain unclear. Here we deleted the SRCR5 domain of CD163 in MARC-145 cells using CRISPR/Cas9 to generate a CD163^ΔSRCR5^ MARC-145 cell line. The modification of CD163 had no impact on CD163 expression. CD163^ΔSRCR5^ cells were completely resistant to infection by PRRSV-2 strains Li11, CHR6, TJM, and VR2332. The modified cells showed no cytokine response to PRRSV-2 infection and maintained normal cell vitality comparable with the WT cells. The resistant phenotype of the cells was stably maintained through cell passages. There were no replication transcription complexes in the CD163^ΔSRCR5^ cells. SRCR5 deletion did not disturb the colocalization of CD163 and PRRSV-N in early endosomes (EE). However, the interaction of the viral proteins GP2a, GP3, or GP5 with CD163, which is involved in virus uncoating was affected. Furthermore, 77 CD163-binding cellular proteins affected by the SRCR5 deletion were identified by LC–MS/MS. Inhibition of calpain 1 trapped the virions in EE and forced then into late endosomes but did not block viral attachment and internalization, suggesting that calpain 1 is involved in the uncoating. Overall, CD163^ΔSRCR5^ MARC-145 cells are fully resistant to PRRSV-2 infection and calpain 1 is identified as a novel host protein that interacts with CD163 to facilitate PRRSV uncoating.

## Introduction

Porcine reproductive and respiratory syndrome (PRRS) is one of the most devastating viral diseases of pigs that has a significant economic impact on the pig industry worldwide. The causative agent, PRRS virus (PRRSV), is a single-stranded, positive-sense RNA virus, belonging to the family Arteriviridae, order Nidovirales (Cavanagh, 1997; Neumann et al., 2005). Its genome is about 15 kb in length and encodes six structural proteins, including the envelope protein E, membrane protein M and glycoproteins (GPs) GP2 (or GP2a), GP3, GP4, and GP5, and a replication and transcription complex (Han and Yoo, 2014).

CD163 is a 130 kDa type-I transmembrane glycoprotein that is exclusively expressed by cells of the monocyte/macrophage lineage (Alex Law et al., 1993; Ritter et al., 1999). The green monkey kidney cell lines (MA-104 and MARC-145) also express CD163 (Calvert et al., 2007). Its extracellular domain is composed of nine SRCR domains of about 100–110 residues arranged in tandem, and is encoded by a separate exon (Backe et al., 1991; Alex Law et al., 1993; Ritter et al., 1999). The well-known function of CD163 is that of a scavenger receptor involved in the clearance of cell-free hemoglobin (Hb) and Hb/haptoglobin complexes (Onofre et al., 2009; Subramanian et al., 2013). The membrane-associated CD163 (mCD163) and its shed form, soluble CD163 (sCD163), share an inverse correlation of occurrence *in vivo*, and are both involved in the clearance of Hb (Davis and Zarev, 2005; Subramanian et al., 2013). The fifth scavenger receptor cysteine-rich domain (SRCR5) of CD163 is identified as the critical domain for PRRSV infection (Van Gorp et al., 2010; Ma et al., 2017).

Calpain is a calcium-dependent neutral protease with two isoenzyme forms, calpain 1 and calpain 2 (Rami, 2003). Growing evidence indicates that the calpains play a critical role in viral infections (Liu et al., 2004). Inhibition of calpain protease activity protects myocardial injury from virus-induced apoptosis (DeBiasi et al., 2001). The non-structural 5A phosphoprotein of the hepatitis C virus is an inducer and a substrate of the calcium-dependent calpain protease(s) (Kalamvoki and Mavromara, 2004). Calpains also have a role in mediating the proteolytic modification of human cytomegalovirus UL112-113 proteins (Wang et al., 2015). Calpain 1 is required for RNA replication of Echovirus 1 and is especially important at a later stage of infection (Upla et al., 2008). However, the role of calpain 1 in PRRSV infection has not been reported.

Through a clathrin-coated vesicle, PRRSV is delivered into early endosomes (EEs) where it interacts with CD163 to release its genome into the cytoplasm (Whitworth and Prather, 2017). The EE is a major sorting station where the contents are either sent back to the plasma membrane or proceed further down to late endosomes and lysosomes (Gruenberg and Van Der Goot, 2006). The lysosomes are a degradative and dead-end compartment for many viruses. For a productive infection, the PRRSV requires trafficking only through CD163-positive EE, and not the late endosomes and lysosomes (Van Gorp et al., 2009). The PRRSV genome in the cytoplasm is translated immediately into replicase polyproteins, that are cleaved into individual non-structural proteins and assembled into replication transcription complexes (RTCs) (Snijder et al., 2013; Burkard et al., 2017). The emergence of the RTCs containing the non-structural protein 2 (nsp2) is a signal of initiating replication of the viral genome and generation of structural proteins (Snijder et al., 2013; Burkard et al., 2017).

Since porcine alveolar macrophages (PAMs) are the main PRRSV host cells *in vivo*, we chose to use them for the PRRSV study to obtain more relevant information like that *in vivo*. However, some of the characteristics of the cells limit their application prospects. For example, cells with edited genes cannot be utilized for follow-up experiments because they are non-proliferative *in vitro*. The MARC-145 cell line is another choice for us to study PRRSV infection *in vitro*. It has been verified that pigs without SRCR5 domain of CD163 are resistant to PRRSV-1 and their macrophages are fully resistant to both PRRSV-1 and PRRSV-2 (Burkard et al., 2017, 2018). However, whether it is possible to substitute the PAMs with MARC-145 cells to test the relation between CD163^ΔSRCR5^ and PRRSV *in vitro*, and how the deletion of the SRCR5 domain confers resistance to CD163^ΔSRCR5^ PAMs to PRRSV infection remain unclear. Therefore, we chose to modify CD163 in MARC-145 cells and then use the modified cells to answer the question mentioned above in this study.

## Materials and Methods

### Cell Culture and Viruses

African green monkey kidney cells MARC-145 and 293T cells were cultured in Dulbecco’s modified Eagle’s medium (DMEM; Sigma, St. Louis, MO, United States) supplemented with 10% fetal bovine serum (FBS; PAA, Pasching, Austria) for multiplication culture or 2% FBS for maintenance culture at 37°C in 5% CO_2_. PRRSV strains CHR6, Li11, TJM, and ATCC VR2332 were provided by Dr. Guihong Zhang of South China Agricultural University. A recombinant PRRSV strain, containing enhanced green fluorescent protein (EGFP) as a specific marker (designated PRRSV-EGFP), was gifted by Dr. Shuqi Xiao from Northwest A&F University, China. The EGFP was inserted between the N protein and 3′-UTR of PRRSV genome (Wang et al., 2013). All the strains were propagated and titrated on MARC-145 cells.

### gRNA Design and Cutting Efficiency Assessment

The gRNAs intended to delete exon 7 of CD163 were designed from the website of http://crispr.mit.edu/ and their RNA sequences are available upon request. Four potential gRNAs named gRNA1, gRNA2 gRNA3, and gRNA4 located in the 300 bp interval of intron 6 terminal sequence and two located in the 101 bp long of intron 7 named gRNA8 and gRNA9 were chosen based on the comprehensive score on the web. gRNAs 1–4 were inserted downstream of the human U6 promoter in pX458R plasmid (Addgene, United States) containing gene sequence expressing Cas9 protein and DsRed protein, and gRNAs 8 and 9 were similarly cloned downstream of the human U6 promoter in pX458 plasmid containing gene sequence expressing Cas9 protein and GFP linked via the ‘self-cleaving’ 2A peptide sequence as previously described (He et al., 2015). Subsequently, pX458 or pX458R carrying an intended gRNA was transfected into MARC-145 cells. After GFP or DsRed positive cells were sorted out from transfected cells using a FACS Aria II cell sorter (Becton Dickinson), the sorted cells were cultured further for 2 days followed by extraction of the genomic DNA (DNeasy Blood & Tissues Kit, Qiagen). PCR was performed across the exon 7 upstream target sites using LsgRNA primers to obtain a 847 bp product, and across the exon 7 downstream target sites using RsgRNA primers to obtain a 726 bp product. These PCR products were used in T7 endonuclease I assay (T7E1, NEB) to assess the cutting efficiency of individual gRNA *in vitro*.

### Monoclonal Cell Culture and Genotype Detection

MARC-145 cells were co-transfected with plasmids encoding gRNA1 and gRNA9. Each individual cell expressing GFP and DsRed double fluorescent proteins was sorted into a well using the FACS Aria II cell sorter and cultured for 50 days. Once monoclonal cell populations emerged, their genomes were extracted for genotype analysis using the DNeasy Blood and Tissue Kit (Qiagen). Primers Co-F and Co-R designed to span intron 6 to 7 were used to amplify a product of 1077 bp length from the intact allele, and a product of 466 bp length if complete deletion of exon 7 had occurred. The PCR products of different lengths were extracted using a DNA gel extraction kit (Omega) and sequenced.

### Quantitative Real-Time Reverse-Transcription Polymerase Chain Reaction (qRT-PCR)

Total RNA was isolated from MARC-145 cells using TRIzol reagent (Invitrogen) and reverse transcribed using the Prime Script RT reagent kit (Takara) according to the manufacturer’s instructions. The RNA levels were measured using SYBR green (Takara) real-time PCR. Relative quantities of CD163 and PRRSV nucleocapsid protein (N) mRNA expression were evaluated using the 2^–ΔCT^ (Ct is the threshold cycle) method. The primers used in the study are listed in Supplementary Table S2.

### Western Blotting

MARC-145 cells were harvested using cell lysis buffer (Beyotime). The lysates were boiled and subjected to electrophoresis on 10% acrylamide gels (Bio-Rad) to separate the proteins and subsequently the proteins were transferred to polyvinylidene difluoride membranes (Millipore). After the membranes were blocked, the bands corresponding to CD163, PRRSV-N protein, and glyceraldehyde phosphate dehydrogenase (GAPDH) were visualized by incubating with antibodies against CD163 (Abcam, ab87099), PRRSV-N protein (Jeno Biotech), and GAPDH (Cell Signaling Technology) overnight at 4°C. Subsequently, the CD163 and GAPDH blots were incubated with HRP-labeled anti-rabbit IgG antibodies (Cell Signaling Technology), and the PRRSV-N blot was incubated with HRP-conjugated anti-mouse IgG antibody (Cell Signaling Technology). All the protein bands were visualized with ECL Plus chemiluminescence reagent (Pierce, Rockford, IL, United States).

### Immunofluorescence and Confocal Microscopy

Cells were fixed with 4% paraformaldehyde for 10 min and then permeabilized with 0.5% Triton X-100 for 15 min. After blocking with 5% BSA, the cells were incubated with the corresponding target protein antibodies overnight at 4°C. The next day the cells were washed with PBS followed by incubation with Alexa Fluor 555-conjugated secondary antibody (Cell Signal Technology) or Alexa Fluor 488-conjugated secondary antibody (Cell Signal Technology) for 1 h. The nuclei were stained with 4′, 6′-diamidino-2-phenylindole (DAPI, Beyotime, China) or cytoskeleton was stained with phalloidin AF568 phalloidin (Sigma). Finally, the cells were observed using a fluorescence microscope (Nikon ECLIPSE Ti2) or a laser scanning confocal microscope (TCS-SP5, LEICA).

### PRRSV Titration Assay

PRRSV titers (the 50% tissue culture infective dose (TCID_50_) per 0.1 ml) were assayed according to the Reed-Muench method. Briefly, 0.1 ml of viral supernatants was mixed with 0.9 ml of fresh DMEM containing 2% FBS and a series of 12 10-fold serial dilutions were made. The 96-well plates seeded with MARC-145 cells were prepared prior to the titration assay. Each gradient diluent was added to the 96-well plates with eight repetitions and the plates were cultured continually at 37°C for monitoring the cytopathic effects.

### Flow Cytometry

Cells were seeded in 12-well plates prior to inoculation with PRRSV for the indicated time periods. Then the cells were digested using trypsin (Sigma) and resuspended in PBS. The harvested cells were detected by FACS on a FACS Calibur (Becton Dickinson) and the data were analyzed using the FlowJo software.

### Plasmid Construction, Transfection, Immunoprecipitation (IP), and Co-IP

The viral GP2a, GP3, GP4, GP5, and PRRSV-N genes from CHR6 strain were amplified by PCR using gene-specific primers (Supplementary Table S2) and cloned into the pmCherry-N1 vector (Takara). The CD163^WT^ and CD163^ΔSRCR5^ genes were cloned into pcDNA 3.1 plasmid containing Myc-His tag by Sanggon Biotech Corporation. These plasmids were transfected into 293T cells using Lipofectamine 3000 (Invitrogen). The transfected cells were harvested using 750 μl of cell lysis buffer (Beyotime) for IP. 200 μl of the cell lysates were stored for western blot assay and the remaining lysates were used for co-IP assays. For the co-IP assays, 60 μl of protein A/G Agarose beads (Merck Millipore) were incubated with a Myc antibody (Cell Signal Technology). Following that, the antibody-bead mixtures were incubated with 0.2M dimethyl pimelimidate. After washing the antibody-bead mixtures with 0.1M boric acid buffer, the cell lysates were mixed with beads and the lysates-bead/antibody conjugate mixtures were incubated at 4°C overnight. The Ag–Ab complexes were eluted from the beads. Western blotting was performed to check for the precipitation of the proteins.

### Protein Identification by Liquid Chromatography-Tandem Mass Spectrometry (LC–MS/MS)

Cells seeded in 100 mm^2^ plates were mock-inoculated or inoculated with CHR6 strain at MOI = 2 for 1 h at 4°C and then placed at 37°C for 30 min. Cells were washed three times in ice-cold PBS and the proteins were harvested as described in the IP and co-IP protocols above. Protein A/G agarose beads were incubated with anti-CD163 antibody (Abcam, ab189915). The harvested proteins samples were separated on a 10% SDS-PAGE gel and stained with Coomassie Brilliant Blue G-250 (Bio-Rad). The trypsin-digested and purified proteins were injected in Thermo Q Exactive^TM^ to identify proteins precipitated by the CD163 antibody. The MS spectra were searched against a custom-made protein database containing the *Chlorocebus sabaeus* CD163 sequence. The data from LC–MS/MS were analyzed using the Blast2GO program to annotate the protein functions and protein classification at UniProt.

### Statistical Analysis

All experiments were repeated at least three independent times. Statistical analyses were performed using SPSS (version 16.0) or GraphPad Prism 5.0. Significant results were analyzed using Student’s *t*-test or one-way analysis of variance (ANOVA). *P* < 0.05 was considered significant.

## Results

### CRISPR/Cas9 Mediated Deletion of SRCR5 of CD163 in MARC-145 Cells

**FIGURE 1:**
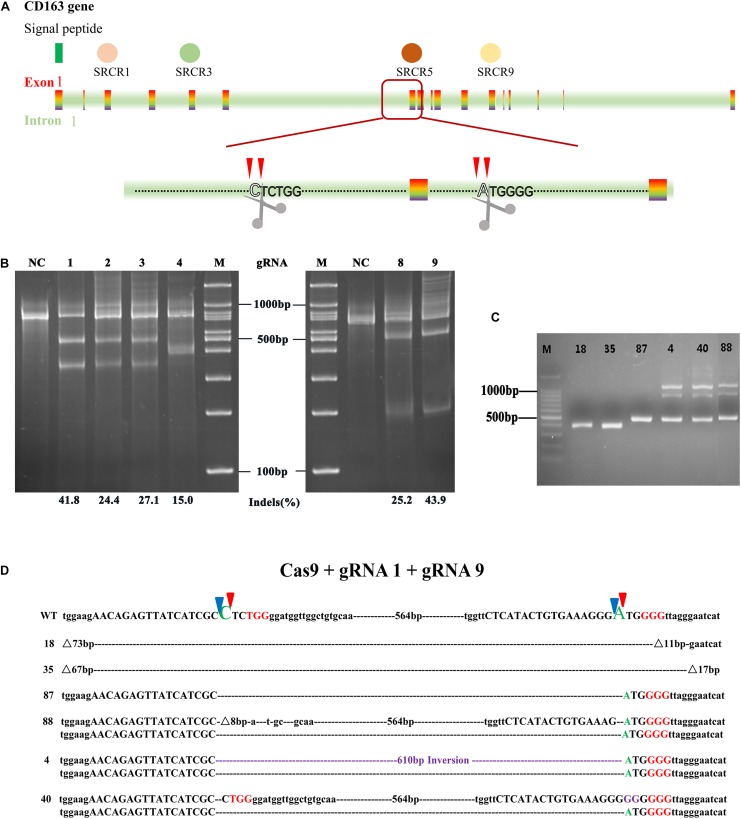
Designation of gRNAs relative to the CRISPR/Cas9 system used to generate an exon 7 deletion of CD163. **(A)** Schematic of CD163 in the green monkey (*Chlorocebus sabaeus*) genome on chromosome 11 (Gene ID: 103218507). Indicated in the colored rectangle are the 17 exons encoding the CD163 mRNA. Indicated in varied colored circles above are the scavenger receptor cysteine-rich (SRCR) domains. Excision of exon 7 using two guide RNAs (gRNA1 and gRNA9) located in the flanking introns results in SRCR5 removal from the encoded protein. **(B)** *In vitro* assessment of the guide RNAs (gRNA1, gRNA2, gRNA3, gRNA4, gRNA8, and gRNA9). MARC-145 cells were transfected with a single plasmid encoding a guide RNA + Cas9. The transfected cells were isolated by FACS through either GFP or DsRed expression. The cutting efficiency of single gRNA was assessed by a T7E1 assay. **(C)** Length of PCR products in MARC-145 monoclonal cells with different genotype. MARC-145 cells were co-transfected with a pair of plasmids encoding gRNA1 or gRNA9 + Cas9. The extracted DNA from the monoclonal cells derived from transfected cells with homozygous genotype (labeled with 18, 35, and 87) and heterozygous genotype (labeled with 4, 40, and 88) were assessed by PCR across intron 6 and exon 8. The unmodified genome PCR was predicted to result in a 1100 bp product, whilst exon 7 deletion would result in a 500 bp PCR product. **(D)** Sanger sequencing results for CD163 from the MARC-145 cell lines. Indicated in red uppercase letters are protospacer adjacent motif (PAM) located in the flanking introns of exon 7, indicated in purple are insertion sequences. Predicted cleavage sites of gRNA are represented by blue and red triangles. Dotted line without a number label represents large fragment deletions at predicted cleavage sites and symbol represents extra deleted fragments flanking the predicted cleavage sites in the CD163 gene. WT, wild type DNA sequence.

To generate a MARC-145 cell line with deletion of the SRCR5 domain of CD163 (CD163^ΔSRCR5^), CRISPR/Cas9 gene editing strategy was used to remove the whole exon 7 encoding SRCR5 in the CD163 gene. As shown in Figure 1A, a couple of guide RNAs (gRNAs) served as molecular scissors flanking exon 7 leading to the precise deletion of exon 7 of CD163 in MARC-145 along with Cas9. Meanwhile, the remaining exons retained the appropriate splicing. Based on this, we designed six unique targeting sequences (crRNA) with a corresponding protospacer adjacent motif (PAM) located in the intron regions flanking exon 7 as candidate gRNAs.

To choose a pair of gRNAs with high cutting efficiency, each of the six sequences inserted in pX458 (gRNA8 and 9)or pX458R (gRNA1, 2, 3, and 4)was transfected into MARC-145 cells *in vitro*. The transfected cells were sorted by fluorescence-activated cell sorting (FACS) for GFP or DsRed, and their genomic DNA was prepared for T7 endonuclease I assay (T7E1) to assess the cutting efficiency at the target site. The results showed that all six gRNAs could functionally induce NHEJ at its target sites and shared efficiencies ranging from 15.0 to 43.9% (Figure 1B). However, only gRNA1 (41.8%) that recognized the upstream intron of exon 7, and gRNA9 (43.9%) that recognized the downstream intron of exon 7, were selected for subsequent experiments because of their relatively higher indels rate.

To investigate whether the co-transfection of gRNA1 and gRNA9 into MARC-145 cells could precisely delete SRCR5, the co-transfected cells were seeded at one cell per well in 96-well plates by FACS using dual fluorescence selection, and cultured until they grew into colonies of monoclonal cells. Genotype identification showed that only three of the monoclonal cells labeled 18, 35, and 87 contained a deletion of the intended size, 54 of the monoclonal cells had undesired deletion sizes, and 38 of the monoclonal cells were wild type (WT) (Figure 1C and Supplementary Figure S1). To further identify the genotype of the monoclonal cells, full length and truncated PCR products of the monoclonal cells labeled number 18, 35, 87, 4, 40, and 88 were sequenced (Figures 1C,D). Only the cells labeled 87 showed an exact deletion and subsequent splicing without indels of random nucleotides at the designed cutting sites, while monoclonal cell lines labeled 18 and 35 showed extra DNA fragments missing at the anticipated cutting sites in addition to the intended deletion. For example, cell line 18 lacked 73 bp at the cutting site of gRNA1 and 11 bp at the site of gRNA9, while cell line 35 was 67 and 17 bp deficient at the corresponding sites, respectively. Cell lines labeled 88 and 40 were found to be heterozygous for the exon 7 with the deletion of one allele and the other allele being WT. Interestingly, cell line 4 was found to be biallelic for the exon 7 with deletion of one allele and the other allele showing inversion of the whole exon 7 DNA fragment between the cutting sites (Figure 1D). Taken together, we identified three homozygous CD163^ΔSRCR5/ΔSRCR5^ MARC-145 cell lines (number 18, 35, and 87), two heterozygous CD163^WT/ΔSRCR5^ cell lines (number 88, and 40), and one biallelic CD163^ΔSRCR5/ΔSRCR5^ cell line (number 4) (Supplementary Table S1).

### CD163^ΔSRCR5^ MARC-145 Cells Show No Susceptibility to PRRSV

To distinguish the susceptibility of the MARC-145 cell lines with different genotypes (homozygous, heterozygous, and biallelic) to PRRSV infection, the cell lines 87, 88, 40, and 4 were infected with PRRSV-EGFP at multiplicity of infection (MOI) of 1, while the WT cells infected with PRRSV-EGFP served as a positive control. The number of infected cells was calculated by flow cytometry at 48 h post-infection (hpi). As illustrated in Supplementary Figure S2A, the cell lines 88 and 40 retained their susceptibility to PRRSV infection like the WT cells. In contrast, cell lines 87 and 4 were found to be highly resistant to PRRSV infection. In addition, RNA was extracted from these cell lines over the time course for quantitative reverse transcription PCR (qRT-PCR) analysis to quantify the viral ORF 7 transcription level. As shown in Supplementary Figure S2B, both cell lines 88 and 40 showed similar viral replication trends over the time course compared with the WT cells. The PRRSV replication amount reached a peak at 24 hpi and then slowly declined over time. On the contrary, virus replication was undetected in the cell lines 87 and 4 during the time course of infection. To rule out the effect of CD163 expression level on the tolerance of the cell lines, we extracted RNA from the different cell lines without PRRSV-EGFP infection. The qRT-PCR result revealed that the CD163 mRNA levels showed no difference between the identified cell lines (Supplementary Figure S2C), which was consistent with the results of the western blot analysis (Supplementary Figure S2D). To further confirm the results, we performed a time course analysis to determine whether the virus could replicate in these cell lines and then infect the neighboring cells. Cell lines inoculated with PRRSV-EGFP at MOI = 1 were firstly monitored by fluorescence microscope at indicated time points, and then harvested for western blot analysis and the supernatant samples were collected for analysis of the 50% tissue culture infective dose (TCID_50_) at the corresponding time points. As shown in Supplementary Figure S3A, PRRSV-EGFP were observed in WT, 88 and 40 at indicated time points, but no GFP, representing virions, was found in cell lines 87 and 4. Furthermore, all cell lines except 87 and 4 developed severe cytopathic effect at 72 hpi. The result of western blotting showed that there was no N protein expression in 87 and 4 cells (Supplementary Figure S3B), which is consistent with the fluorescence data. In addition, TCID_50_ analysis revealed that the viral infectivity produced from the WT cells was significantly different compared to cell lines 87 and 4 that do not support virus production. However, cell lines 88 and 40 displayed a strong ability for productive infection like the WT cells (Supplementary Figure S3C). In summary, the heterozygous genotype MARC-145 cell lines 88 and 40 displayed high susceptibility to PRRSV infection, while the homozygous and biallelic cell lines 87 and 4 were fully resistant to PRRSV infection. This difference in susceptibility to the virus was independent of CD163 expression, but associated with SRCR5 domain.

### CD163^ΔSRCR5^ Cells Are Resistant to PRRSV-2 Isolates Infection

Previous studies have shown that macrophages with CD163 SRCR5 deletion derived from peripheral blood monocyte are not susceptible to infection with PRRSV-2 (Burkard et al., 2017). To investigate whether CD163 SRCR5 deletion confers resistance to 87 and 4 cells to various subtypes of PRRSV-2, WT, 87 and 4 cells were infected with the highly pathogenic strains (Li11 and TJM) and classic strains (CHR6 and VR2332) at MOI = 1 for 48 h. Cells were harvested to extract viral RNA for qRT-PCR analysis. As shown in Supplementary Figures S4A–D (left panel), the mRNA levels of ORF 7 from the four viral strains were significantly lower in 87 and 4 than in the WT cells. A small amount of ORF 7 mRNA was detected in the 87 and 4 cells which may be the result of internalization and storage of the virus, rather than the replication of the virus in the cells. To verify the hypothesis, the cells inoculated with different PRRSV strains at MOI = 1 were harvested at 48 hpi and the expression of PRRSV-N protein was assessed by western blotting. As expected, different subtypes of the PRRSV-N protein were clearly detected in the WT cells. However, no PRRSV-N protein was detected in 87 and 4 cells (Supplementary Figures S4E,F), which suggests that 87 and 4 cell lines are not susceptible to infection by PRRSV-2. To further confirm the results, supernatants from cells inoculated with the different PRRSV strains at MOI = 1 were collected at 48 hpi for TCID_50_ assay. As shown in Supplementary Figures S4A–D (right panel), the infectious virions produced from the WT cells were significantly higher than that from 87 to 4 cells. Taken together, CD163-edited cell lines 87 and 4 were resistant to infection with PRRSV-2 isolates.

### CD163^ΔSRCR5^ Cells Show No Cytokine Response Post PRRSV Infection

**FIGURE 2:**
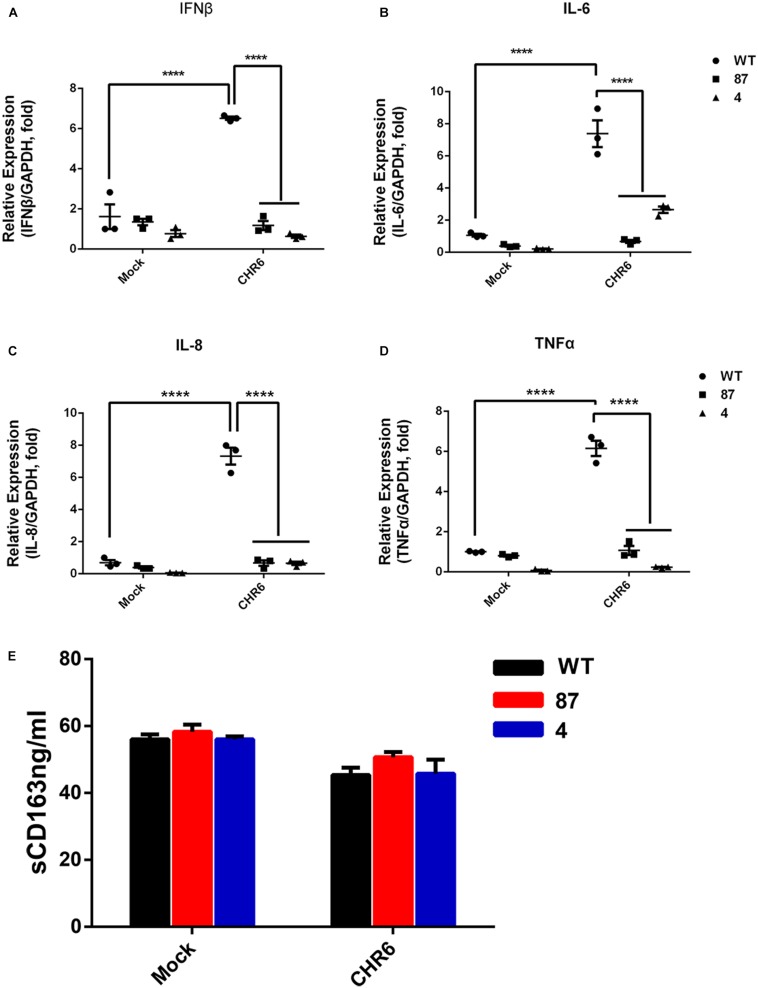
Gene-edited cell lines show no cytokine response to PRRSV infection and generally normal cytokine levels. **(A–D)** mRNA were collected from WT, 87, and 4 cells mock-inoculated or inoculated with PRRSV CHR6 strain for 0 or 48 h. Cytokine levels of IFN-β **(A)**, IL-6 **(B)**, IL-8 **(C)**, and TNF-α **(D)** were quantified by qRT-PCR assay. Error bars represent standard deviation (*n* = 3). Statistical analysis was performed using one-way ANOVA and an unpaired *t*-test. ^∗∗∗∗^*P* < 0.0001. **(E)** Cellular supernatants collected from WT, 87, and 4 at 0 or 48 hpi were assessed for the level of soluble CD163 (sCD163) using a commercial ELISA (*n* = 3). Statistical analysis using an unpaired *t*-test showed no significant difference.

Since CD163, as a soluble factor, exhibits cytokine-driven functions, we measured the expression of sCD163 and cytokines in the gene-edited cell lines 87 and 4. First, the cell lines were inoculated or mock-inoculated with CHR6 at MOI = 1, and then the supernatants were harvested at 48 hpi. Subsequently, the sCD163 level was measured in the supernatants using a commercially available enzyme-linked immunosorbent assay kit (ELISA). As shown in Figure 2E, in the mock-infected group, sCD163 levels were calculated to be 56.1 ± 3.3 ng/ml in WT cells and 57.2 ± 6.4 ng/ml in 87 and 4 cells. In infected group, sCD163 levels in the WT cells ranged between 42.7 and 52.0 ng/ml with a median value of 45.37 ng/ml, and in the gene-edited cells were at a median value of 48.2 ng/ml with a range of 41.1 to 57.1 ng/ml. This demonstrates that there is no difference in the sCD163 level between the WT and gene-edited cells in either the infected or mock-infected group. The mRNA expression analysis of cytokines in cells was carried out using the same protocol as described above for the ELISA assay. As shown in Figures 2A–D, cytokines such as TNF-α, IFN-β, IL-6, and IL-8 in the mock group showed no significant difference between WT cells and gene-modified cells. Furthermore, we found that the mRNA expression of these cytokines increased significantly at 48 hpi when the WT cells were inoculated with CHR6, while the effects of PRRSV on the expression of the cytokines did not change significantly in the gene-edited cells, which means that there was no inflammatory response in modified cells infected with the virus.

### CD163^ΔSRCR5^ Cells Maintain Normal Cell Vitality and Retain PRRSV Resistance After Cell Passages

**FIGURE 3:**
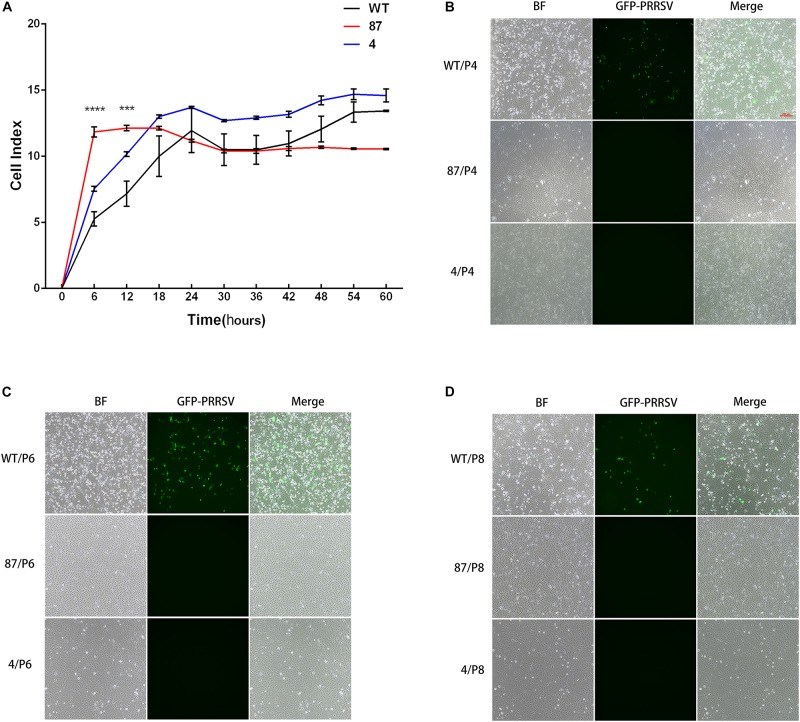
Gene-edited cell lines 87 and 4 maintain normal cell vitality and still fully resist PRRSV infection after cell passage. **(A)** Real-time monitoring of cell growth and vitality through the xCELLigence system (ACEA Biosciences, Inc., San Diego, CA, United States). A total of 10,000 cells of WT, 87, and 4 were seeded in an electronic microtiter plate and the cell index were continuously monitored by the xCELLigence system for 72 h. Statistical analysis was performed using an unpaired *t*-test (^∗∗∗^*P* < 0.001; ^∗∗∗∗^*P* < 0.0001). **(B–D)** Gene-edited cell lines 87 and 4 confer resistance to PRRSV after increasing the cell passages. Cells from WT, 87, and 4 at 4th **(B)**, 6th **(C)**, and 8th **(D)** cell passages were inoculated with PRRSV-EGFP (MOI = 1) for 48 h. Cells were then observed by fluorescence microscope (Bar: 200 μm).

To confirm that the CD163 SRCR5 deletion does not affect cell growth, real-time growth and vitality of cells were monitored using the xCELLigence system (Martinez-Serra et al., 2014). Cell vitality analysis revealed that there were significant differences in 87 and WT cells during the first 12 h. When they reached 18 h and progress to 60 h, the vitality levels of the three cell lines were basically the same over time and the cells reached a plateau during the last period (54–60 h) (Figure 3A). To confirm that the gene-edited cells maintain the characteristic of virus resistance, the anti-PRRSV property of different cell passages were tested (Ke et al., 2011). As expected, the green fluorescence representing virus replication was clearly observed in WT but not in 87 and 4 cells after cell passages for 4, 6, or 8 generations (named P4, P6, and P8) at 48 hpi (Figures 3B–D). Collectively, these findings demonstrated that the deletion of CD163 SRCR5 domain did not affect the growth of the modified cells and that their anti-virus property is independent of cell passages.

### PRRSV Virions Are Arrested Before the Formation of RTCs in CD163^ΔSRCR5^ Cells

**FIGURE 4:**
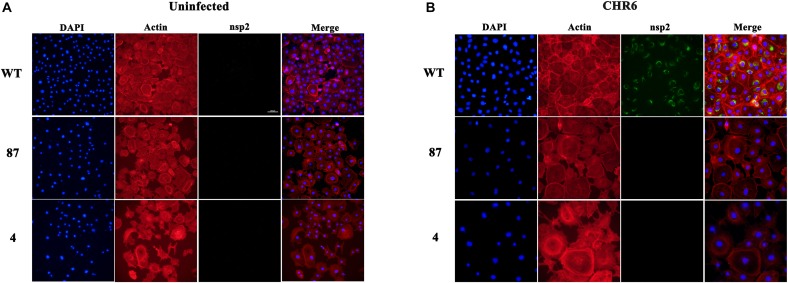
Anti-PRRSV property of gene-edited cell lines 87 and 4 is due to the inhibition of the formation of replication transcription complex (RTC). **(A,B)** MARC-145 cells from WT (top panel), 87 (middle panel), and 4 (bottom panel) cell lines were mock-inoculated **(A)** or inoculated **(B)** with CHR6 strain (MOI = 1) for 24 h and then fixed for immunofluorescent staining of PRRSV-nsp2 protein (green), DAPI (blue), and phalloidin (red). Scale bar represents 200 μm.

As the viral genomes are transported into the cytoplasm, they initiate the translation process, and subsequently RTCs are assembled. The RTCs existing in the cells represent the initiation of viral replication (Snijder et al., 2013; Burkard et al., 2017). The absence of replicated mRNA of ORF 7 and lack of expression of PRRSV-N protein in the virus-infected CD163^ΔSRCR5^ cells may be due to the fact that the virions are arrested before the formation of RTCs. To confirm this hypothesis, we inoculated WT, 87 and 4 cells with CHR6 at MOI = 2 for 24 h. Cells were then stained for PRRSV Nsp2 following permeabilization. As shown in Figures 4A,B, no RTCs were observed in infected 87 and 4 cells. However, many RTCs were present in the WT cells. These data confirm that PRRSV failed to start productive infection and the infection was arrested before the formation of RTCs in CD163^ΔSRCR5^ MARC-145 cells.

### SRCR5 Deletion Blocks Virus Uncoating

**FIGURE 5:**
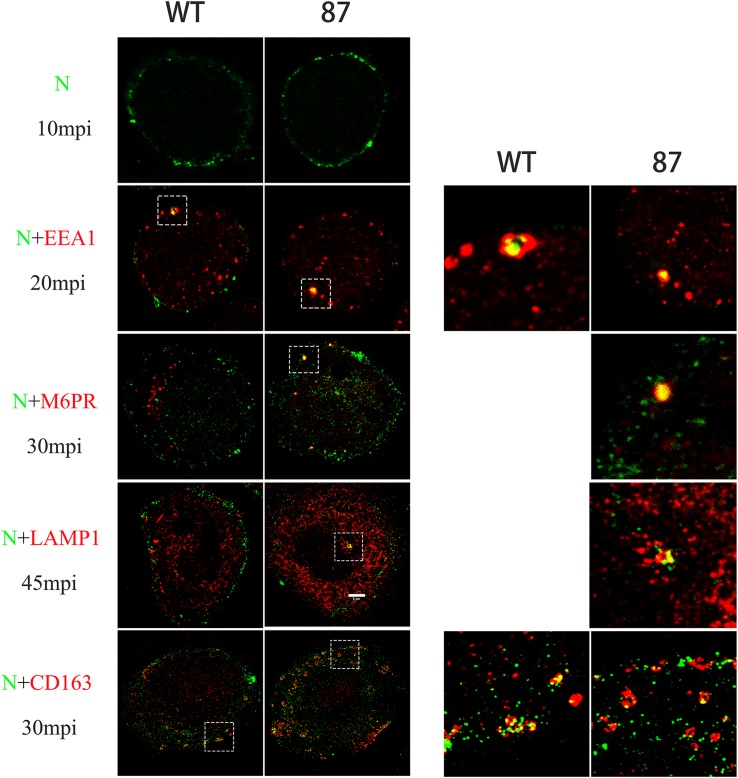
Colocalization between CHR6 virions and different marker identifying a specific compartment in the endocytic pathway or CD163 receptor of WT and CD163^ΔSRCR5^ cells. Cells were inoculated with CHR6 strain (MOI = 1) at 4°C for 1 h and subsequently incubated for different time points at 37°C. Cells were then fixed and permeabilized with 0.5% Triton X-100 for immunofluorescent staining with a mouse anti-PRRSV N antibody (green), a rabbit anti-CD163 antibody (red), and antibodies to different compartments of the endocytic pathway (red). Early endosome antigen 1 (EEA1) was stained to label early endosomes, the cation-independent mannose-6-phosphate receptor (CI-M6PR) was stained to label late endosomes, and lysosome-associated membrane protein 1 (Lamp1) was stained to label lysosomes. Representative co-localization (yellow) images are shown. Scale bar represents 5 μm.

Before the formation of RTCs, the virions stay in the EE where CD163 meets virions to facilitate uncoating (Van Gorp et al., 2009). Therefore, the failure of formation of RTCs may have been caused by the deletion of SRCR5 in CD163 that arrests the virions in the EE. To determine whether SRCR5 deletion inhibits PRRSV uncoating in the EE, colocalization studies were conducted in WT and 87 cells to visualize PRRSV and markers of endocytic compartments, early endosomes (EEA1), late endosomes (CI-M6PR), and lysosomes (Lamp1). As depicted in Figure 5, PRRSV was found at the cell surface and just beneath the plasma membrane of the WT and 87 cells after incubation for 10 min with CHR6 strain. The colocalization of the virions and EE were visualized at 20 min in WT and 87 cells. However, the colocalization of virions and late endosomes or lysosomes was observed only in the 87 cells. These data indicate that CD163 SRCR5 deletion does not affect PRRSV attachment and internalization, but blocks virions uncoating in the EE to inhibit the genome release into the cytoplasm, subsequently virions are transported to the later step of endocytic pathway, and finally were degraded in the lysosomes.

To investigate whether SRCR5 deletion affects virus uncoating in EE by disturbing the colocalization of CD163 and virus, immunofluorescence confocal assay of PRRSV-N and CD163 was performed, as described above. Interestingly, PRRSV was found to colocalize with CD163 in the EE in both WT and 87 cells infected with CHR6 for 30 min (Figure 5). These data indicate that SRCR5 deletion has no influence on the interaction between PRRSV and CD163 in the EE.

### SRCR5 Deletion Interferes With the Interaction of Viral GP2a, GP3, and GP5 With CD163

**FIGURE 6:**
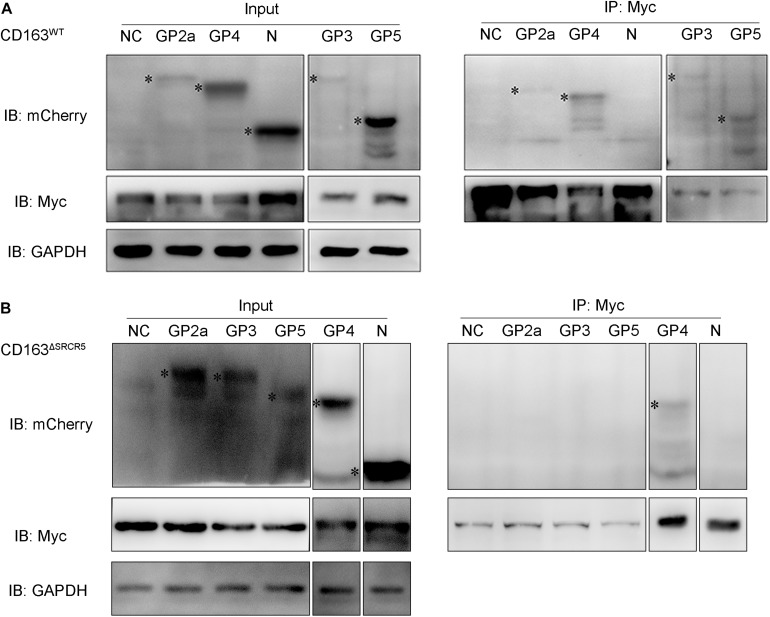
The interaction of CD163^WT^ or CD163^ΔSRCR5^ with PRRSV GPs and PRRSV-N. **(A,B)** 293T cells were transfected with control plasmids as negative control or plasmids encoding individual viral envelope GPs or PRRSV-N in combination with pcDNA3.1 vector expressing CD163^WT^ **(A)** or CD163^ΔSRCR5^ **(B)** protein. Cell lysate was immunoprecipitated with an anti-Myc monoclonal antibody. The viral GPs and N proteins were detected by 10% SDS-PAGE using anti-mCherry and anti-Myc antibodies. The identified target protein bands are labeled with black asterisk on the left side of each lane.

It was previously reported that the viral envelope proteins GP2a and GP4 specifically interact with CD163 expressed in BHK-21 cells (Das et al., 2010). Since SRCR5 deletion does not affect the recognition of the virus to the CD163^ΔSRCR5^ receptor, it may affect the interaction between the viral envelope GPs and CD163 in the EE to interfere with the uncoating of the virus. To test this hypothesis, CD163^WT^ or CD163^ΔSRCR5^ were co-expressed along with each of the four envelope GPs (GP2a, GP3, GP4, and GP5) in 293T cells, and the interactions were examined by coimmunoprecipitation (co-IP) with porcine anti-Myc monoclonal antibody. The results showed that Myc (representing CD163^WT^) monoclonal antibody was able to co-IP the four envelope GPs (GP2a, GP3, GP4 and GP5) separately in multiple repeat experiments (Figure 6A). However, when the individual GPs were co-expressed with CD163^ΔSRCR5^, the Myc (representing CD163^ΔSRCR5^) monoclonal antibody could only co-IP the GP4 protein in repeat experiments (Figure 6B). We also examined the interaction between PRRSV-N and CD163 because of their obvious co-localization in the EE. The PRRSV-N protein could not be detected by immunoprecipitation with Myc antibody (CD163^WT^ and CD163^ΔSRCR5^) in the co-transfected cells (Figure 6). These results suggest that in addition to the known interactions of GP4 and GP2a with CD163, GP3 and GP5 also interact with the CD163 receptor. More importantly, SRCR5 deletion interferes with the interaction of viral GP2a, GP3, and GP5 with CD163.

### Calpain 1 Plays a Vital Role in Virus Uncoating

**FIGURE 7:**
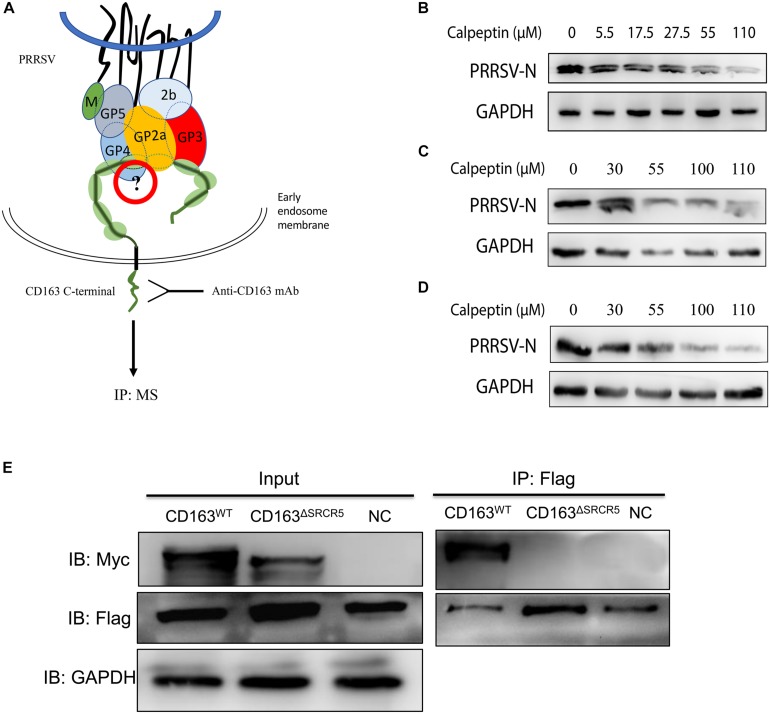
Identification of host cellular proteins that interact with CD163^WT^ but not CD163^ΔSRCR5^ after PRRSV infection. **(A)** A strategy for identifying the cellular CD163-binding proteins associated with the SRCR5 domain. Cell lines WT and 87 were mock-inoculated or inoculated with CHR6 strain at MOI = 2 for 1 h at 4°C and then placed at 37°C for 30 min. Cells were then harvested for co-IP assay. Protein A/G agarose beads were incubated with an anti-CD163 antibody. The harvested proteins samples were separated on a 10% SDS-PAGE gel and stained with Coomassie Brilliant Blue G-250 for LC–MS/MS. **(B–D)** Calpain 1 inhibitor calpeptin blocks PRRSV replication both in MARC-145 cells and PAMs. PAMs **(B)** and MARC-145 cells **(C)** were infected with CHR6 (MOI = 1) in the presence of calpain 1 inhibitor calpeptin at concentration gradients for 36 h at 37°C or MARC-145 cells **(D)** were pretreated with calpeptin at concentration gradients for 1 h and then inoculated with CHR6 strain at MOI = 1 at 37°C for 36 h. Expression of PRRSV N protein was examined by western blotting to confirm the inhibitory effect of the inhibitor on PRRSV replication. **(E)** 293T cells were transfected with control plasmids as negative control or plasmids encoding CD163^WT^ or CD163^ΔSRCR5^ together with pcDNA3.1 expressing calpain 1 protein. Cell lysate was immunoprecipitated with an anti-Flag monoclonal antibody. The CD163^WT^ and CD163^ΔSRCR5^ were detected by 10% SDS-PAGE using an anti-Myc antibody.

It has been reported that CD163 homologs from divergent mammalian species can functionally replace porcine CD163 in several cell lines (Calvert et al., 2007), which suggests that in addition to CD163, there may be other cell host proteins that play a decisive role in virus uncoating. Based on this, we hypothesized that there might exist some unknown CD163-binding cellular proteins that take part in virus uncoating. To determine whether SRCR5 deletion has an impact on the interaction between CD163 and CD163-binding proteins of host cells, LC–MS/MS was performed to identify the CD163-binding host proteins in WT and 87 cells (Figure 7A). LC–MS/MS data showed that the number of host proteins in virus-infected WT and 87 cells were more than that of the corresponding uninfected cells (Supplementary Figure S5), which indicates that some of the host proteins are required for virus infection. We further calculated the number of cellular proteins that were present only in the infected WT cells, but not in the infected 87, uninfected WT or uninfected 87 cells (labeled with 0010). As shown in Supplementary Figure S5, there were 77 proteins identified in infected WT cells but not in other three groups which may interact with CD163 SRCR5 during virus uncoating (Supplementary File S1).

The GO annotation results showed that calpain 1 protein is a calcium-dependent cysteine-type protease (Supplementary Files S2, S3) and the number of experiments (found/total) in CRAPome database for calpain 1 is 19/411 (Mellacheruvu et al., 2013). It has been reported that calpain 1 has a role in the infection and uncoating of echovirus 1 and coxsackievirus (Upla et al., 2008; Bozym et al., 2010). Based on this, we focused on calpain 1 for further studies. To test whether calpain 1 is involved in virus infection, MARC-145 cells or PAMs were infected with PRRSV in the presence or absence of the calpain 1 inhibitor, calpeptin (Upla et al., 2008). As shown in Figures 7B–D, the expression of PRRSV N protein decreased both in the MARC-145 cells and PAMs treated with calpeptin compared to that in the mock-treated cells and the effect of inhibition was dose-dependent, suggesting that calpain 1 is associated with PRRSV replication. To test the interaction of calpain 1 with CD163, co-IP assay was performed. As shown in Figure 7E, calpain 1 interacted with CD163 but not CD163^ΔSRCR5^, suggesting that the interaction of calpain 1 with CD163 requires the SRCR5 domain, which further demonstrates the data of LC–MS/MS.

**FIGURE 8:**
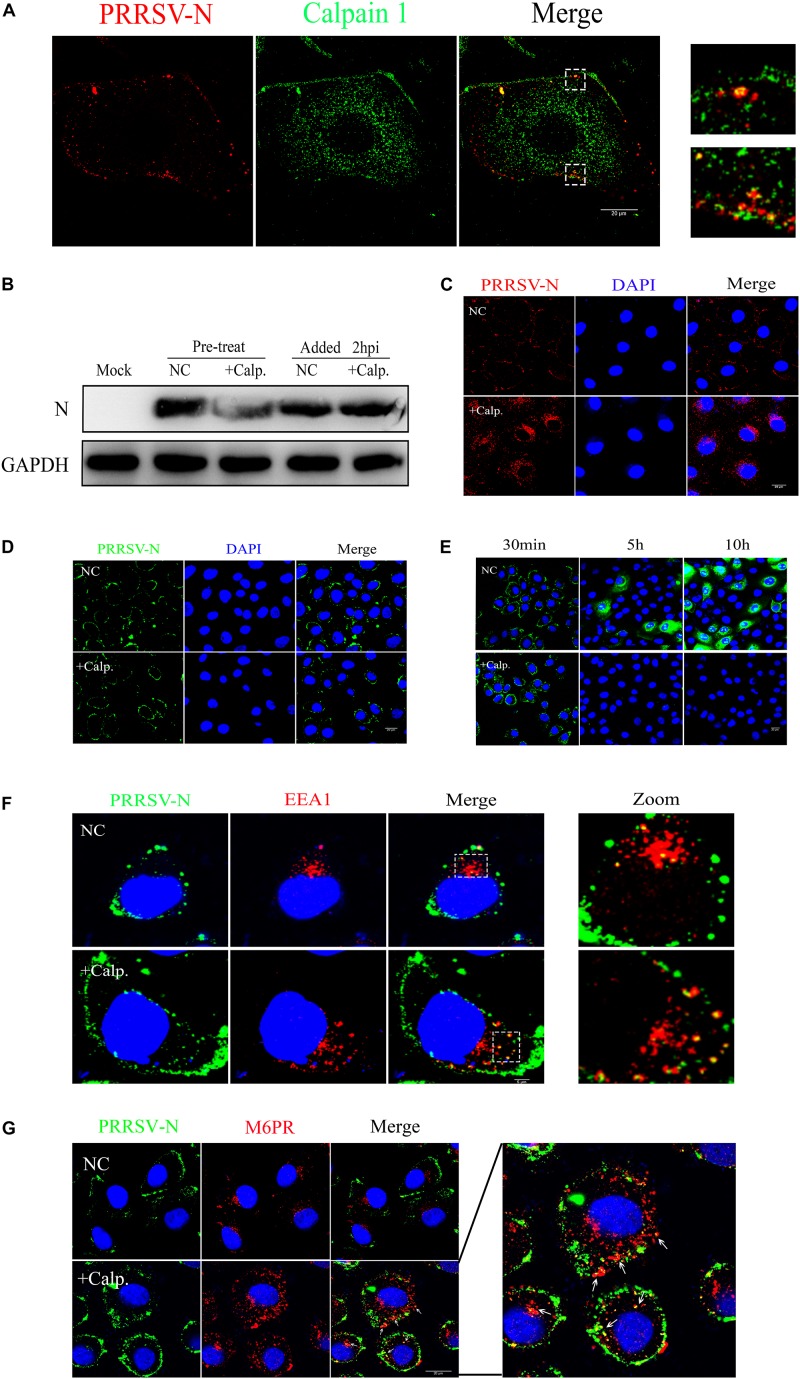
Calpain 1 is required for PRRSV uncoating. **(A)** Co-localization of calpain 1 (Abcam, ab108400) and PRRSV-N. WT cells were fixed after CHR6 infection for 30 min, and subjected to fluorescence confocal experiments to detect calpain 1 (Green) and PRRSV-N (Red). Scale bar represents 20 μm. **(B)** Calpain 1 is required for events occurring before virus internalization. WT cells were infected with CHR6 for 2 h and then treated with calpain 1 inhibitor calpeptin (110 μM) for 24 h or pretreated with calpeptin for 2 h prior to virus infection for 24 h. Expression of PRRSV N protein was examined by western blotting to confirm the inhibitory effect of calpeptin on PRRSV replication. **(C)** An immunofluorescence-based assay for viral location in infected WT cells treated with DMSO as a negative control (NC) or calpeptin (calp., 110 μM) for 2 h at 37°C. Red fluorescence indicates virus and blue fluorescence indicates nucleus (DAPI). Scale bar represents 20 μm. **(D,E)** Viral attachment assay was performed in WT cells exposed to CHR6 (MOI = 1) for 1 h at 4°C in the presence of DMSO (NC) or calpeptin (110 μM) **(D)**, and viral internalization and replication assays were performed in cells infected with CHR6 (MOI = 1) for 30 min (viral internalization phase), 5 h, or 10 h at 37°C in the presence of DMSO (NC) or calpeptin (110 μM) **(E)**. Green fluorescence indicates absorbed virus, internalized virus or replicated virus, and blue fluorescence indicates nucleus (DAPI). Scale bar represents 20 μm. **(F,G)** Colocalization between CHR6 virions and the markers of early endosomes (EEA1) **(F)** and late endosomes (CI-M6PR) **(G)**. Calpeptin-treated or DMSO-treated cells were inoculated with CHR6 strain (MOI = 1) for 1 h at 4°C and subsequently incubated for 30 min **(F)** or 45 min **(G)** at 37°C. Green fluorescence indicates virus located in cells, red fluorescence indicates early endosomes compartment (EEA1) or late endosomes (M6PR) and blue fluorescence indicates nucleus (DAPI). Yellow indicates colocalization (arrowheads) between EEA1 or M6PR with PRRSV-N. Scale bar represents 20 μm.

Since an obvious effect of calpain 1 on virus replication was observed, we next determined how the protein participated in suppressing virus infection. Firstly, using a fluorescence-based assay for colocalization that discriminates between calpain 1 and PRRSV-N in MARC-145 cells exposed to CHR6 strain for 30 min, we found that calpain 1 and PRRSV-N colocalized (Figure 8A), which indicates that calpain 1 may be involved in viral replication directly. To understand which event of virus infection was associated with calpain 1, cells were infected with CHR6 for 2 h and then treated with calpain 1 inhibitor calpeptin for 24 h or pretreated with calpeptin for 2 h prior to virus infection for 24 h. As shown in Figure 8B, the expression of PRRSV-N protein was significantly reduced in the pretreated cells compared to that in the untreated cells. However, calpeptin lost its inhibitory effect on PRRSV-N expression when added at a post-entry time point (added 2 hpi, Figure 8B), indicating that the role of calpain 1 in viral infection is specific to early events of the infection. Cells were next pretreated with calpeptin for 2 h and then inoculated with CHR6 for 2 h. As shown in Figure 8C, many virions were significantly blocked to a perinuclear compartment by calpeptin treatment compared to the untreated cells, suggesting that calpain 1 is required for the trafficking of the virus particles and it may participate in virus uncoating. To confirm the role of calpain 1 along with the CD163 receptor in mediating PRRSV uncoating in MARC-145 cells, viral attachment assay was performed in cells exposed to CHR6 (MOI = 1) for 1 h at 4°C in the presence of DMSO or calpeptin (Figure 8D). Viral internalization and replication assays were performed in cells infected with CHR6 (MOI = 1) for 30 min (viral internalization phase), 5 h, or 10 h at 37°C in the presence of DMSO or calpeptin (Figure 8E). Compared to DMSO-treated cells, calpeptin specifically inhibited virus replication while having no effect on viral attachment and entry into treated cells (Figures 8D,E). In addition, we observed an obvious colocalization between EE marker (EEA1) and PRRSV-N in virus-infected cells that were pretreated with calpeptin or not (Figure 8F), which indicates that calpain 1 functions in the EE with CD163 and calpain 1 inhibitor has no effect on the internalization of the virions. Further studies were done using confocal microscopy to colocalize PRRSV-N and late endosomes marker M6PR in cells pretreated with calpeptin for 2 h (or not) and then exposed to CHR6 at 37°C for 45 min. As shown in Figure 8G, the addition of calpeptin forced the virions into late endosomes (arrowheads), downstream of endocytosis, while in the cells without the addition of calpeptin, no late endosomes containing virions were observed. These data suggest that calpain 1 is necessary for virus uncoating in the EE. Overall, these results demonstrate that SRCR5 deletion interferes with the interaction of CD163 with calpain 1 which plays a vital role in virus uncoating.

## Discussion

Two regions of SRCR5 have been reported to be involved in PRRSV infection: loop 5–6 and the ligand-binding pocket (LBP) (Welch and Calvert, 2010). Multiple binding sites on the outside of CD163 are important for its interaction with PRRSV. The SRCR5-9 Fc protein of CD163 shows an additive anti-PRRSV activity due to its binding to PRRSV virions (Chen et al., 2014). Our previous study demonstrates that pigs with a partial deletion of the CD163 SRCR5 domain including LBP confer resistance to PRRSV-2 infection *in vivo* and *in vitro* (Guo et al., 2019). In the current study, we generated a MARC-145 cell line with CD163^ΔSRCR5^ phenotype using a CRISPR/Cas9 method. The results of this study showed that CD163^ΔSRCR5^ cells expressed CD163 normally, and maintained the normal growth vitality like the WT cells (Figure 3). The viral challenge experiments showed that the gene-edited cells were fully resistant to PRRSV-2 isolates such as Li11, CHR6, TJM, and VR2332 (Supplementary Figure S4), and retained the anti-PRRSV property stably over several cell passages (not just the 8 passages recorded, data not shown) (Figure 3). In CD163^ΔSRCR5^ cells infected with Li11 and TJM, small amounts of active virions were detected in recycled supernatants that could infect other cells continuously. They may be the original virions that were added and not newly produced ones (Supplementary Figure S4). However, in cells infected with CHR6 and VR2332, no active viruses were detected in the recycled supernatants (Supplementary Figure S4). Further, the concentration of sCD163 in the supernatants, and the mRNA expression of cytokines such as IFN-β, IL-6, IL-8, and TNF-α in the CD163-modified cells did not change before and after the viral infection (Figure 2), which is consistent with the results reported by Burkard et al. (2017). However, we did not determine its susceptibility to PRRSV-1 and whether the cell line possesses PRRSV-1 resistance is unknown. Further research is needed to explore it in the future. Overall, these data suggest that it is feasible to substitute CD163^ΔSRCR5^ PAMs with CD163^ΔSRCR5^ MARC-145 cells to study the effect of CD163^ΔSRCR5^ in PRRSV infection *in vitro*.

A previous study demonstrated that the PRRSV infection process is arrested prior to the formation of the RTCs in CD163^ΔSRCR5^ PAMs (Burkard et al., 2017). Consistent with this, we observed that no RTCs were detectable in virus-infected CD163^ΔSRCR5^ MARC-145 cells (Figure 4), which shows that the arrest in infection of CD163^ΔSRCR5^ cells occurs prior to viral replication. Van Gorp et al. (2009) showed that PRRSV is not transported down to the late endosomes after it moves into the EE in PAMs and interacts with CD163 in the EE. However, in our study, colocalization results showed that the virus colocalized with markers of EE, late endosomes, and lysosomes in CD163^ΔSRCR5^ MARC-145 cells (Figure 5).

Our results of co-IP analysis showed that SRCR5 deletion interfered with the interaction of viral GP2a, GP3, and GP5 with CD163 (Figure 6). The interference between CD163 and viral GPs may cause the inhibition of PRRSV uncoating in the EE, thus conferring resistance of CD163^ΔSRCR5^ cells to PRRSV-2 infection. The LC–MS/MS data confirmed our hypothesis that several CD163-binding proteins in the host cells interact with the SRCR5 domain of CD163 to facilitate virus uncoating in the EE. A total of 77 cellular CD163-binding proteins that were affected by the SRCR5 deletion were identified by LC–MS/MS. Calpains are ubiquitous and involved in many different cellular functions (Bozym et al., 2010). They are capable of remodeling the cytoskeleton (Upla et al., 2008), which suggests that calpains may play a key role in vesicular trafficking and viral entry and intracellular migration. Furthermore, based on previous studies that reported an important role for calpain 1 in infection, intracellular trafficking and uncoating of echovirus 1, coxsackievirus, human immunodeficiency virus type 1, hepatitis C virus and herpes simplex virus type 1 (Upla et al., 2008; Bozym et al., 2010; Zheng et al., 2014), we focused on calpain 1 for further studies and found that inhibition of calpain 1 strongly suppressed viral replication in both MARC-145 cells and PAMs (Figure 7). The calpain 1 – CD163 interaction requires the SRCR5 domain, which is consistent with our data of LC–MS/MS. The virions are trapped in the EE in the SRCR5 deleted cells, and forced down into late endosomes and lysosomes by calpeptin treatment. Calpeptin inhibited PRRSV infection by blocking uncoating of the virions. Moreover, we found that calpain 1 protein colocalized with PRRSV-N in the cells at 30 min post-infection (mpi), and its function was independent of viral attachment and internalization into the EE (Figure 8). These results indicate that calpain 1 is involved in PRRSV uncoating. We will further focus on it using a modified cell line with the deletion of calpain 1 and PAMs, the natural host cells of PRRSV in pigs. In addition, the role of the other cellular proteins identified in PRRSV infection will also be investigated in the future.

**FIGURE 9:**
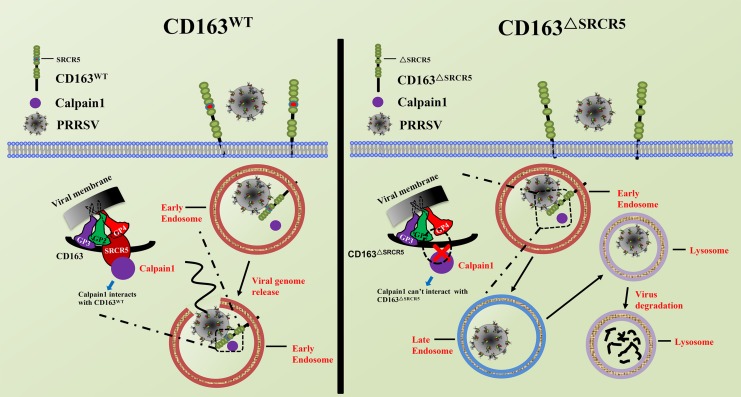
Schematic model of PRRSV life cycle in CD163^WT^ and CD163^ΔSRCR5^ cells. In CD163^WT^ cells, after virions internalize into early endosomes, viral glycoproteins (GP2a, 3, 4, and 5) interact with the CD163 receptor and host proteins such as calpain 1, thus facilitating virus uncoating from early endosomes to release genome to the cytoplasm. Subsequently, the viral genome initiates its replication. However, after virions traffic to early endosomes and meet with CD163^ΔSRCR5^ in CD163^ΔSRCR5^ cells, only viral GP4 interacts with the CD163^ΔSRCR5^ receptor and calpain 1 fails to interact with SRCR5 domain. Due to the failed interaction among CD163^ΔSRCR5^, viral glycoproteins and calpain 1, virus uncoating is blocked in the early endosomes and virions are forced to the late endosomes and finally to the lysosomes, where they are degraded.

In summary, as illustrated in Figure 9, after the virions are internalized into the EE in CD163^WT^ cells, the viral GPs (GP2a, 3, 4, and 5) interact with CD163 and the cellular protein, calpain 1, to facilitate uncoating to release the genome. Subsequently, the viral genome initiates its replication. However, in CD163^ΔSRCR5^ cells, only viral GP4 interacts with CD163^ΔSRCR5^. Due to the failure of interaction among CD163^ΔSRCR5^, viral GPs, and calpain 1, virus uncoating is blocked and the virions are forced into the late endosomes and finally to lysosomes, where they are degraded. Calpain 1 is identified as a novel protein associated with viral infection that interacts with CD163 to facilitate PRRSV uncoating in the EE. Calpain 1 is a promising target for PRRSV prevention and control in future.

## Data Availability Statement

All datasets generated for this study are included in the article/Supplementary Material.

## Author Contributions

PY and CG conceived and designed the study and contributed to the interpretation of the data and took part in the critical revision of the manuscript. PY, RW, and WD performed the experiments, analyzed the data, and drafted the manuscript. PY, RW, WD, ZZ, XZ, YC, XL, and CG coordinated the study and read and approved the final manuscript.

## Conflict of Interest

The authors declare that the research was conducted in the absence of any commercial or financial relationships that could be construed as a potential conflict of interest.

## Supplementary Material

FIGURE S1 Genotyping of MARC-145 monoclonal cells. MARC-145 cells were co-transfected with a combination of two plasmids encoding a gRNA1 or gRNA9 plus Cas9. Individual cell with GFP and DsRed expression was isolated and seeded into a well of 96-well plates by FACS Aria II cell sorter, and then the sorted cells were cultured to acquire monoclonal cells. The collected genome of each monoclonal cells was amplified using primers Co-F and Co-R designed to span intron 6 to 7 of CD163 gene. The amplified length of CD163 WT is predicted to a 1100 bp product, whilst exon 7 deletion should result in a 500 bp PCR product. The red rectangle represents the monoclonal cells containing an intended deletion of exon7. “M” represents DNA marker and “W” represents MARC-145 cells without transfection.

FIGURE S2 CD163^ΔSRCR5^ cells are not susceptible to infection with PRRSV-EGFP and show comparable levels of CD163 protein and mRNA as the WT cells. **(A)** WT and gene-edited MARC-145 cell lines were mock-inoculated or inoculated with PRRSV-EGFP at MOI = 1 for 48 h and the infected cells were detected by flow cytometer. **(B)** Gene-edited and WT MARC-145 cell lines were inoculated with PRRSV-EGFP (MOI = 1) and then harvested for qRT-PCR analysis of PRRSV-N expression at 12, 24, 36, 48, 60, and 72 hpi. **(C,D)** mRNA and proteins were extracted from WT and gene-edited MARC-145 cells and CD163 mRNA expression was assessed by qRT-PCR **(C)** and CD163 protein level was assessed by immunoblotting analysis with quantitation of densitometry for CD163 **(D)**. Statistical analysis was performed using an unpaired t-test for the WT cells against gene-edited cell lines. Significant differences in the results compared to the WT are indicated by ^∗^*P* < 0.05, ^∗∗^*P* < 0.01, and ^∗∗∗^*P* < 0.001. Error bars represent SEM, *n* = 3.

FIGURE S3 MARC-145 cells with deletion of CD163 SRCR5 show complete resistance to PRRSV infection. **(A,B)** MARC-145 cell lines were inoculated with PRRSV-EGFP (MOI = 1) for the indicated time points. Cells were observed by fluorescence microscope (Bar, 100 μm) **(A)**. Simultaneously, cells were harvested for the detection of PRRSV-N expression by immunoblotting analysis **(B)**. **(C)** Replication growth curves of PRRSV-EGFP. Cells were inoculated with PRRSV at MOI = 1. Cell supernatants were collected at indicated time points to measure the released viral particles by TCID_50_ analysis. Significant differences in results compared to the WT are indicated as follows: ^∗^*P* < 0.05, ^∗∗^*P* < 0.01, ^∗∗∗^*P* < 0.001, and ^*⁣*⁣**^*P* < 0.0001. Error bars represent SEM, *n* = 3.

FIGURE S4 Gene-edited cell lines 87 and 4 are not susceptible to infection with PRRSV-2. **(A–F)** MARC-145 cells from WT, 87, and 4 were inoculated with PRRSV-2 strains Li11, CHR6, TJM, and VR2332 at MOI = 1 for 48 h, and mRNA was extracted for qRT-PCR analysis (**A–D**, left panel). PRRSV-N mRNA expression were statistically analysed using an unpaired t-test of WT cells against 87 or 4 cells. Simultaneously, cell supernatants were collected to measure the produced infectious particles by TCID_50_ analysis (**A–D**, right panel) and cells were harvested for immunoblotting analysis (**E,F**). Error bars represent SEM, *n* = 3. Significant differences in the results compared to the WT are indicated as follows: ^∗^*P* < 0.05, ^∗∗^*P* < 0.01, ^∗∗∗^*P* < 0.001, and ^*⁣*⁣**^*P* < 0.0001.

FIGURE S5 Data statistics of CD163-binding cellular proteins identified by LC-MS/MS. WT and 87 cells were mock-inoculated or inoculated with CHR6 (MOI = 2) at 4°C for 1 h and then switched to 37°C for 30 min. After cells were harvested, CD163-binding cellular proteins were immunoprecipitated by CD163 antibody (ab189915, Abcam). The “0010” represents CD163-binding proteins of which only identified in CHR6-infected WT cells. The “V” represents PRRSV.

TABLE S1 Genotype and phenotype prediction for CD163 from monoclonal MARC-145 cell line.

TABLE S2 The sequences of primers used in this study.

FILE S1 Statistic analysis of GO annotation of LC-MS/MS data.

FILE S2 Identification of CD163-binding proteins by LC-MS/MS.

FILE S3 Annotation of CD163-binding proteins identified by LC-MS/MS.

## References

[B1] Alex Law S. K. Micklem K. J. Shaw J. M. Zhang X. P. Dong Y. Willis A. C. (1993). A new macrophage differentiation antigen which is a member of the scavenger receptor superfamily. *Eur. J. Immunol.* 23 2320–2325. 10.1002/eji.1830230940 8370408

[B2] Backe E. Schwarting R. Gerdes J. Ernst M. Stein H. (1991). Ber-MAC3: new monoclonal antibody that defines human monocyte/macrophage differentiation antigen. *J. Clin. Pathol.* 44 936–945. 10.1136/jcp.44.11.936 1721628 PMC496636

[B3] Bozym R. A. Morosky S. A. Kim K. S. Cherry S. Coyne C. B. (2010). Release of intracellular calcium stores facilitates coxsackievirus entry into polarized endothelial cells. *PLoS Pathog.* 6:e1001135. 10.1371/journal.ppat.1001135 20949071 PMC2951373

[B4] Burkard C. Lillico S. G. Reid E. Jackson B. Mileham A. J. Ait-Ali T. (2017). Precision engineering for PRRSV resistance in pigs: macrophages from genome edited pigs lacking CD163 SRCR5 domain are fully resistant to both PRRSV genotypes while maintaining biological function. *PLoS Pathog.* 13:e1006206. 10.1371/journal.ppat.1006206 28231264 PMC5322883

[B5] Burkard C. Opriessnig T. Mileham A. J. Stadejek T. Ait-Ali T. Lillico S. G. (2018). Pigs lacking the scavenger receptor cysteine-rich domain 5 of CD163 are resistant to porcine reproductive and respiratory syndrome virus 1 infection. *J. Virol.* 92:e00415-18. 10.1128/JVI.00415-18 29925651 PMC6069206

[B6] Calvert J. G. Slade D. E. Shields S. L. Jolie R. Mannan R. M. Ankenbauer R. G. (2007). CD163 expression confers susceptibility to porcine reproductive and respiratory syndrome viruses. *J. Virol.* 81 7371–7379. 10.1128/JVI.00513-07 17494075 PMC1933360

[B7] Cavanagh D. (1997). Nidovirales: a new order comprising Coronaviridae and Arteriviridae. *Arch. Virol.* 142 629–633. 9349308

[B8] Chen Y. Guo R. He S. Zhang X. Y. Xia X. L. Sun H. C. (2014). Additive inhibition of porcine reproductive and respiratory syndrome virus infection with the soluble sialoadhesin and CD163 receptors. *Virus Res.* 179 85–92. 10.1016/j.virusres.2013.11.008 24246307

[B9] Das P. B. Dinh P. X. Ansari I. H. de Lima M. Osorio F. A. Pattnaik A. K. (2010). The minor envelope glycoproteins GP2a and GP4 of porcine reproductive and respiratory syndrome virus interact with the receptor CD163. *J. Virol.* 84 1731–1740. 10.1128/JVI.01774-09 19939927 PMC2812361

[B10] Davis B. H. Zarev P. V. (2005). Human monocyte CD163 expression inversely correlates with soluble CD163 plasma levels. *Cytometry B Clin. Cytom.* 63 16–22. 10.1002/cyto.b.20031 15624200

[B11] DeBiasi R. L. Edelstein C. L. Sherry B. Tyler K. L. (2001). Calpain inhibition protects against virus-induced apoptotic myocardial injury. *J. Virol.* 75 351–361. 10.1128/JVI.75.1.351-361.2001 11119604 PMC113928

[B12] Gruenberg J. Van Der Goot F. G. (2006). Mechanisms of pathogen entry through the endosomal compartments. *Nat. Rev. Mol. Cell Biol.* 7 495–504. 10.1038/nrm1959 16773132

[B13] Guo C. H. Wang M. Zhu Z. B. He S. Liu H. B. Liu X. F. (2019). Highly efficient generation of pigs harboring a partial deletion of the CD163 SRCR5 domain, which are fully resistant to porcine reproductive and respiratory syndrome virus 2 infection. *Front. Immunol.* 10:1846. 10.3389/Fimmu.2019.01846 31440241 PMC6694839

[B14] Han M. Yoo D. (2014). Engineering the PRRS virus genome: updates and perspectives. *Vet. Microbiol.* 174 279–295. 10.1016/j.vetmic.2014.10.007 25458419 PMC7172560

[B15] He Z. Y. Shi X. Du B. Z. Qin Y. F. Cong P. Q. Chen Y. S. (2015). Highly efficient enrichment of porcine cells with deletions induced by CRISPR/Cas9 using dual fluorescence selection. *J. Biotechnol.* 214 69–74. 10.1016/j.jbiotec.2015.07.011 26200831

[B16] Kalamvoki M. Mavromara P. (2004). Calcium-dependent calpain proteases are implicated in processing of the hepatitis C virus NS5A protein. *J. Virol.* 78 11865–11878. 10.1128/JVI.78.21.11865-11878.2004 15479828 PMC523276

[B17] Ke N. Wang X. Xu X. Abassi Y. A. (2011). The xCELLigence system for real-time and label-free monitoring of cell viability. *Methods Mol. Biol.* 740 33–43. 10.1007/978-1-61779-108-6_6 21468966

[B18] Liu X. L. Van Vleet T. Schnellmann R. G. (2004). The role of calpain in oncotic cell death. *Ann. Rev. Pharmacol. Toxicol.* 44 349–370. 10.1146/annurev.pharmtox.44.101802.121804 14744250

[B19] Ma H. F. Jiang L. G. Qiao S. L. Zhi Y. B. Chen X. X. Yang Y. Y. (2017). The crystal structure of the fifth scavenger receptor cysteine-rich domain of porcine CD163 reveals an important residue involved in porcine reproductive and1 respiratory syndrome virus infection. *J. Virol.* 91:e01897-16. 10.1128/JVI.01897-16 27881657 PMC5244331

[B20] Martinez-Serra J. Gutierrez A. Munoz-Capo S. Navarro-Palou M. Ros T. Amat J. C. (2014). xCELLigence system for real-time label-free monitoring of growth and viability of cell lines from hematological malignancies. *Onco Targets Ther.* 7 985–994. 10.2147/OTT.S62887 24959085 PMC4061162

[B21] Mellacheruvu D. Wright Z. Couzens A. L. Lambert J. P. St-Denis N. A. Li T. (2013). The CRAPome: a contaminant repository for affinity purification-mass spectrometry data. *Nat. Methods* 10 730–736. 10.1038/nmeth.2557 23921808 PMC3773500

[B22] Neumann E. J. Kliebenstein J. B. Johnson C. D. Mabry J. W. Bush E. J. Seitzinger A. H. (2005). Assessment of the economic impact of porcine reproductive and respiratory syndrome on swine production in the United States. *J. Am. Vet. Med. Assoc.* 227 385–392. 10.2460/javma.2005.227.385 16121604

[B23] Onofre G. Karolina M. Jankovičová K. Krejsek J. (2009). Scavenger receptor CD163 and its biological functions. *Acta Medica (Hradec Kralove)* 52 57–61. 10.14712/18059694.2016.105 19777868

[B24] Rami A. (2003). Ischemic neuronal death in the rat hippocampus: the calpain-calpastatin-caspase hypothesis. *Neurobiol. Dis.* 13 75–88. 10.1016/S0969-9961(03)00018-04 12828932

[B25] Ritter M. Buechler C. Langmann T. Schmitz G. (1999). Genomic organization and chromosomal localization of the human CD163 (M130) gene: a member of the scavenger receptor cysteine-rich superfamily. *Biochem. Biophys. Res. Comun.* 260 466–474. 10.1006/bbrc.1999.0866 10403791

[B26] Snijder E. J. Kikkert M. Fang Y. (2013). Arterivirus molecular biology and pathogenesis. *J. Gen. Virol.* 94 2141–2163. 10.1099/vir.0.056341-0 23939974

[B27] Subramanian K. Du R. Tan N. S. Ho B. Ding J. L. (2013). CD163 and IgG codefend against cytotoxic hemoglobin via autocrine and paracrine mechanisms. *J. Immunol.* 190 5267–5278. 10.4049/jimmunol.1202648 23589619

[B28] Upla P. Marjomaki V. Nissinen L. Nylund C. Waris M. Hyypia T. (2008). Calpain 1 and 2 are required for RNA replication of echovirus 1. *J. Virol.* 82 1581–1590. 10.1128/JVI.01375-07 18032503 PMC2224425

[B29] Van Gorp H. Van Breedam W. Delputte P. L. Nauwynck H. J. (2009). The porcine reproductive and respiratory syndrome virus requires trafficking through CD163-positive early endosomes, but not late endosomes, for productive infection. *Arch. Virol.* 154 1939–1943. 10.1007/s00705-009-0527-1 19885719

[B30] Van Gorp H. Van Breedam W. Van Doorsselaere J. Delputte P. L. Nauwynck H. J. (2010). Identification of the CD163 protein domains involved in infection of the porcine reproductive and respiratory syndrome virus. *J. Virol.* 84 3101–3105. 10.1128/JVI.02093-09 20032174 PMC2826032

[B31] Wang C. B. Huang B. C. Kong N. Li Q. Y. Ma Y. P. Li Z. J. (2013). A novel porcine reproductive and respiratory syndrome virus vector system that stably expresses enhanced green fluorescent protein as a separate transcription unit. *Vet. Res.* 44:104. 10.1186/1297-9716-44-104 24176053 PMC4176086

[B32] Wang S. K. Jiang M. J. Lin S. R. Chen M. Y. Wang H. H. Duh C. Y. (2015). Calpains mediate the proteolytic modification of human cytomegalovirus UL112-113 proteins. *J. Gen. Virol.* 96 1115–1126. 10.1099/vir.0.000040 25564485

[B33] Welch S. K. W. Calvert J. G. (2010). A brief review of CD163 and its role in PRRSV infection. *Virus Res.* 154 98–103. 10.1016/j.virusres.2010.07.018 20655964

[B34] Whitworth K. M. Prather R. S. (2017). Gene editing as applied to prevention of reproductive porcine reproductive and respiratory syndrome. *Mol. Reprod. Dev.* 84 926–933. 10.1002/mrd.22811 28390179

[B35] Zheng K. Xiang Y. F. Wang Q. L. Jin F. J. Chen M. Y. Ma K. Q. (2014). Calcium-signal facilitates herpes simplex virus type 1 nuclear transport through slingshot 1 and calpain-1 activation. *Virus Res.* 188 32–37. 10.1016/j.virusres.2014.03.016 24670325

