# Supplementary material for: CD163ΔSRCR5 MARC-145 Cells Resist PRRSV-2 Infection via Inhibiting Virus Uncoating, Which Requires the Interaction of CD163 With Calpain 1
Source: Front Microbiol. 2020 Jan 13;10:3115. doi: 10.3389/fmicb.2019.03115 (PMC6990145; doi:10.3389/fmicb.2019.03115)
Supplement: FIGURE S1 — Genotyping of MARC-145 monoclonal cells. MARC-145 cells were co-transfected with a combination of two plasmids encoding a gRNA1 or gRNA9 plus Cas9. Individual cell with GFP and DsRed expression was isolated and seeded into a well of 96-well plates by FACS Aria II cell sorter, and then the sorted cells were cultured to acquire monoclonal cells. The collected genome of each monoclonal cells was amplified using primers Co-F and Co-R designed to span intron 6 to 7 of CD163 gene. The amplified length of CD163 WT is predicted to a 1100 bp product, whilst exon 7 deletion should result in a 500 bp PCR product. The red rectangle represents the monoclonal cells containing an intended deletion of exon7. “M” represents DNA marker and “W” represents MARC-145 cells without transfection. [file Data_Sheet_1.pdf]

Supplementary Material

1 Supplementary Figures and Tables

1.1 Supplementary Figures

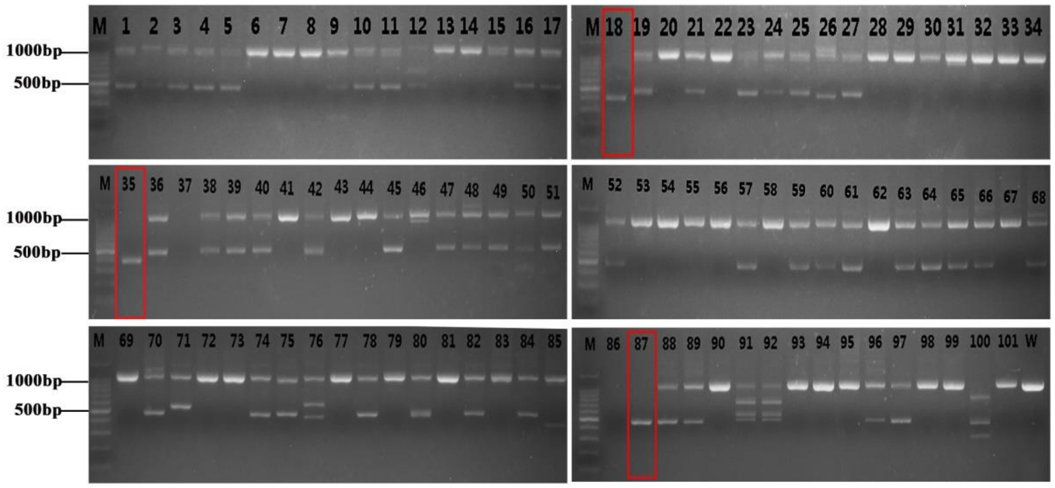

Figure S1 Genotyping of MARC-145 monoclonal cells. MARC-145 cells were co-transfected with a combination of two plasmids encoding a gRNA1 or gRNA9 plus Cas9. Individual cell with GFP and DsRed expression was isolated and seeded into a well of 96-well plates by FACS Aria II cell sorter, and then the sorted cells were cultured to acquire monoclonal cells. The collected genome of each monoclonal cells was amplified using primers Co-F and Co-R designed to span intron 6 to 7 of CD163 gene. The amplified length of CD163 WT is predicted to a 1100 bp product, whilst exon 7 deletion should result in a 500 bp PCR product. The red rectangle represents the monoclonal cells containing an intended deletion of exon7. “M” represents DNA marker and “W” represents MARC-145 cells without transfection.

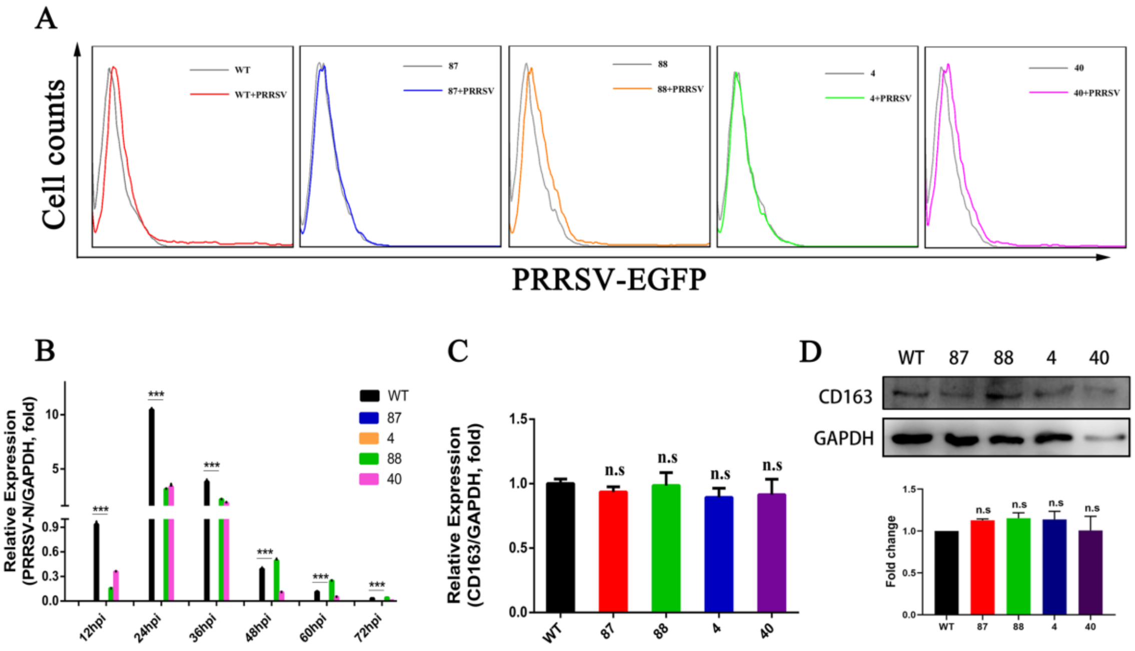

Figure S2 CD163$^{\Delta SRCR5}$ cells are not susceptible to infection with PRRSV-EGFP and show comparable levels of CD163 protein and mRNA as the WT cells. (**A**) WT and gene-edited MARC-145 cell lines were mock-inoculated or inoculated with PRRSV-EGFP at MOI = 1 for 48 h and the infected cells were detected by flow cytometer. (**B**) Gene-edited and WT MARC-145 cell lines were inoculated with PRRSV-EGFP (MOI = 1) and then harvested for qRT-PCR analysis of PRRSV-N expression at 12, 24, 36, 48, 60, and 72 hpi. (**C** and **D**) mRNA and proteins were extracted from WT and gene-edited MARC-145 cells and CD163 mRNA expression was assessed by qRT-PCR (**C**) and CD163 protein level was assessed by immunoblotting analysis with quantitation of densitometry for CD163 (**D**). Statistical analysis was performed using an unpaired t-test for the WT cells against gene-edited cell lines. Significant differences in the results compared to the WT are indicated by *($P < 0.05$), **($P < 0.01$) and ***($P < 0.001$). Error bars represent SEM, n = 3.

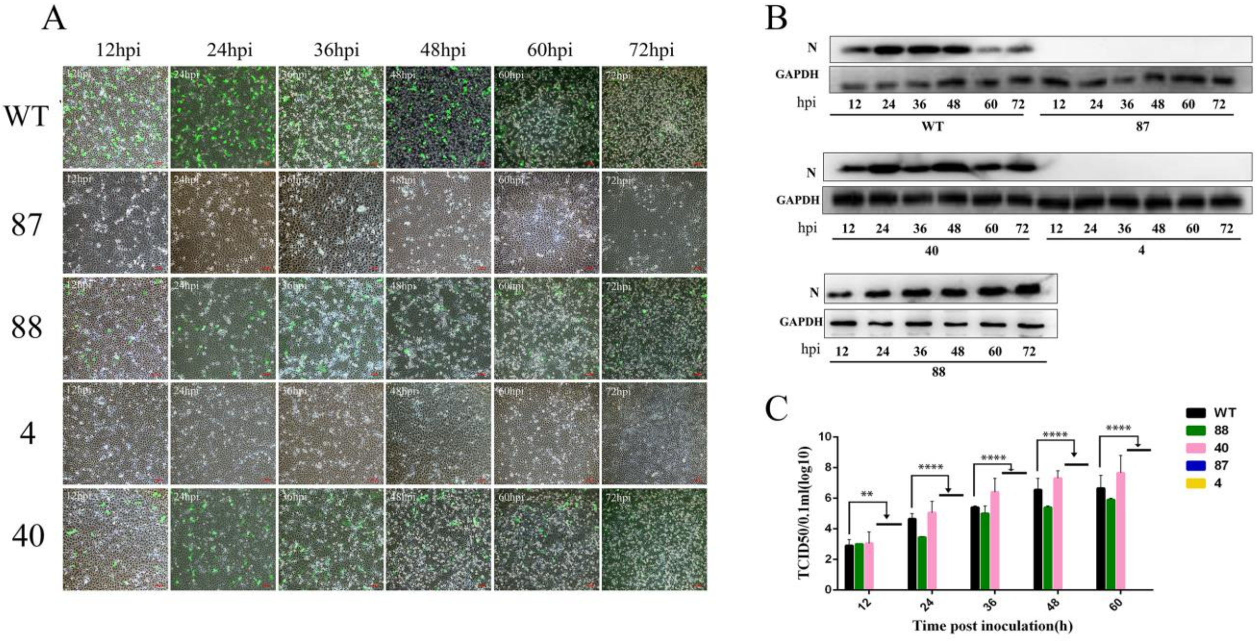

Figure S3 MARC-145 cells with deletion of CD163 SRCR5 show complete resistance to PRRSV infection. (**A** and **B**) MARC-145 cell lines were inoculated with PRRSV-EGFP (MOI = 1) for the indicated time points. Cells were observed by fluorescence microscope (Bar, 100 μm) (**A**). Simultaneously, cells were harvested for the detection of PRRSV-N expression by immunoblotting analysis (**B**). (**C**) Replication growth curves of PRRSV-EGFP. Cells were inoculated with PRRSV at MOI = 1. Cell supernatants were collected at indicated time points to measure the released viral particles by $TCID_{50}$ analysis. Significant differences in results compared to the WT are indicated as follows: *, P < 0.05; **, P < 0.01; ***, P < 0.001; ****, P < 0.0001. Error bars represent SEM, n = 3.

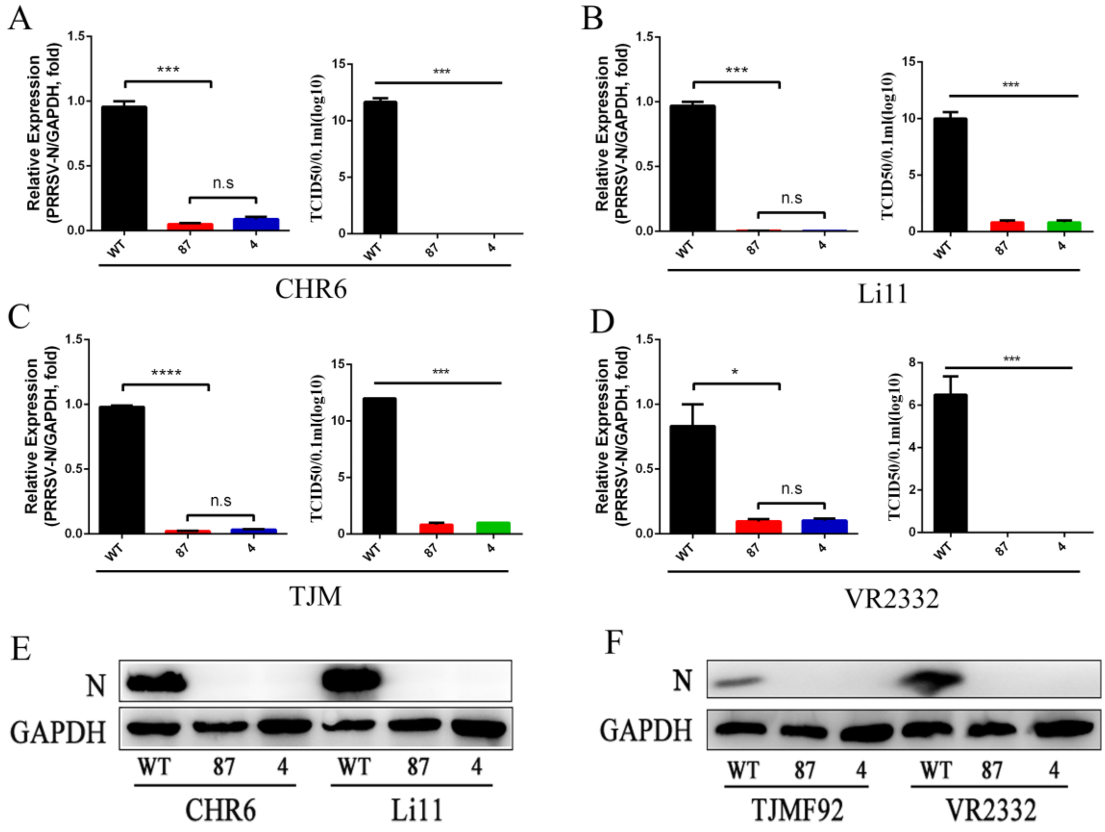

Figure S4 Gene-edited cell lines 87 and 4 are not susceptible to infection with PRRSV-2. (**A** - **F**) MARC-145 cells from WT, 87, and 4 were inoculated with PRRSV-2 strains Li11, CHR6, TJM, and VR2332 at MOI = 1 for 48 h, and mRNA was extracted for qRT-PCR analysis (**A** - **D**, left panel). PRRSV-N mRNA expression were statistically analysed using an unpaired t-test of WT cells against 87 or 4 cells. Simultaneously, cell supernatants were collected to measure the produced infectious particles by $TCID_{50}$ analysis (**A** - **D**, right panel) and cells were harvested for immunoblotting analysis (**E** and **F**). Error bars represent SEM, n = 3. Significant differences in the results compared to the WT are indicated as follows: *, P < 0.05; **, P < 0.01; ***, P < 0.001; ****, P < 0.0001.

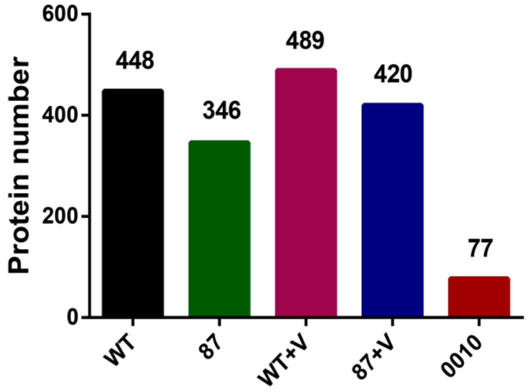

Figure S5 Data statistics of CD163-binding cellular proteins identified by LC-MS/MS. WT and 87 cells were mock-inoculated or inoculated with CHR6 (MOI = 2) at 4 ˚C for 1 h and then switched to 37 ˚C for 30 min. After cells were harvested, CD163-binding cellular proteins were immunoprecipitated by CD163 antibody (ab189915, Abcam). The “0010” represents CD163-binding proteins of which only identified in CHR6-infected WT cells. The “V” represents PRRSV.

1.2 Supplementary Tables

Table S1 Genotype and phenotype prediction for CD163 from monoclonal MARC-145 cell line

Cell No.	Genotype	Size of INDELs	Description	Protein translation	Premature stop codon
18	Homozygous	694bp deletion	Expected deletion of exon7 and extra 73bp deletion on left of gRNA1 target site & 11bp deletion on right of gRNA9 target site.	$CD163^{\Delta SRCR5/\Delta SRCR5}$	NO
35	Homozygous	694bp deletion	Expected deletion of exon7 and extra 67bp deletion on left of gRNA1 target site & 17bp deletion on right of gRNA9 target site.	$CD163^{\Delta SRCR5/\Delta SRCR5}$	NO
87	Homozygous	610bp deletion	Expected deletion of exon7 at designed cutting sites.	$CD163^{\Delta SRCR5/\Delta SRCR5}$	NO
4	Biallelic	610bp inversion; 610bp deletion	One allele: unexpected inversion of 610bp including exon7<br>One allele: deletion of exon 7	$CD163^{\Delta SRCR5/\Delta SRCR5}$	NO
88	Heterozygous	Complicated indels; 610bp deletion	One allele: wild type with complicated indels at cutting sites;<br>Another allele: deletion of exon 7.	$CD163^{WT/\Delta SRCR5}$	NO
40	Heterozygous	Wild type; 609bp deletion	One allele: wild type with 2bp deletion and 2bp insertion at cutting sites;<br>Another allele: deletion of exon 7.	$CD163^{WT/\Delta SRCR5}$	NO

Table S2 The sequences of primers used in this study

Primer group and name	Sequence (5' – 3')
Primers for the CD163 after single gRNA transfection	
LsgRNA-F	GCAGATGAGAAGGTTACG
LsgRNA-R	AGATGAGAAGGTTACGGA
RsgRNA-F	TACCCAACTCAAGGCAGA
RsgRNA-R	ACCACCTTCCTCACCCCTC
Primers for the CD163 after double gRNAs transfection	
Co-F	ATGGTACAAAGAAGGGTCA
Co-R	AGGGCAACTCCACATTTA
Primers for the plasmid construction	
GP2a-F	GGGGTACCGCCACCATGAAATGGGGTCTATGCAA
GP2a-R	CGGGATCCCGCCGTGAGTTCAAAAGAAAAATTGC
GP3-F	CGGGGTACCGCCACCATGGCTAATAGCTGTACA
GP3-R	CGCGGATCCTCGCCGTGCGGCACTGAGAGCTTT
GP4-F	CGGAATTCGCCACCATGGCTGCGCCCCTTTT
GP4-R	CGGGATCCCGAATTGCCAGTAGGATTGCAA
GP5-F	CCGGAATTCGCCACCATGTTGGGGAAGTGCTTGACC
GP5-R	CGGGATCCCGGGGACGACCCCATAGTTCCG
N-F	CGGGATCCATGCCAAATAACAACGGCAAG
N-R	GCTCTAGATCATGCTGAGGGTGATGC
Primers for qRT-PCR	
GAPDH-F	TGACAACAGCCTCAAGATCG
GAPDH-R	GTCTTCTGGGTGGCAGT GAT
mCD163-F2	AGGAGCCCTCATGATAAATC
mCD163-R2	ACCATTCCAGAAGAAAGTGC
mIL-6-F	AGAGGCACTGGCAGAAAAC
mIL-6-R	TGCAGGAACTGGATCAGGAC
mIL-8-F	AGGACAAGAGCCAGGAAGAA
mIL-8-R	ACTGCACCTTCACACAGAGC
mTNF-α-F	TCTGTCTGCTGCACTTTGGAGTGA
mTNF-α-R	TTGAGGGTTTGCTACAACATGGGC

mIFN-β-F	GCAATTGAATGGAAGGCTTGA
mIFN-β-R	CAGCGTCCTCCTTCTGGAACT
