# Supplementary material for: CD163ΔSRCR5 MARC-145 Cells Resist PRRSV-2 Infection via Inhibiting Virus Uncoating, Which Requires the Interaction of CD163 With Calpain 1
Source: Front Microbiol. 2020 Jan 13;10:3115. doi: 10.3389/fmicb.2019.03115 (PMC6990145; doi:10.3389/fmicb.2019.03115)
Supplement: FILE S1 — Statistic analysis of GO annotation of LC-MS/MS data. [file Data_Sheet_2.zip › File S1.pdf]

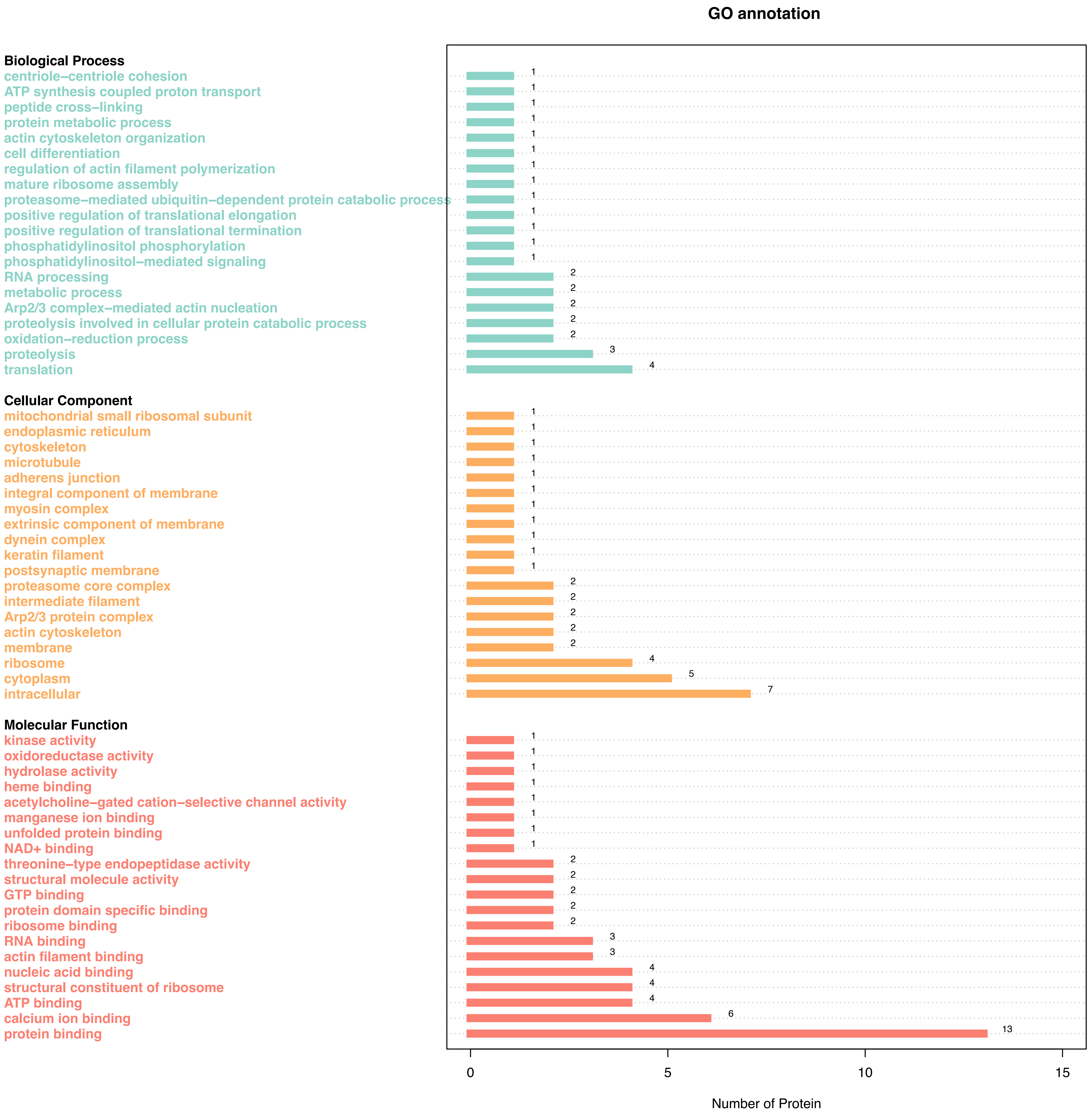
GO annotation
Biological Process
centriole–centriole cohesion 1
ATP synthesis coupled proton transport 1
peptide cross–linking 1
protein metabolic process 1
actin cytoskeleton organization 1
cell differentiation 1
regulation of actin filament polymerization 1
mature ribosome assembly 1
proteasome–mediated ubiquitin–dependent protein catabolic process 1
positive regulation of translational elongation 1
positive regulation of translational termination 1
phosphatidylinositol phosphorylation 1
phosphatidylinositol–mediated signaling 1
RNA processing 2
metabolic process 2
Arp2/3 complex–mediated actin nucleation 2
proteolysis involved in cellular protein catabolic process 2
oxidation–reduction process 2
proteolysis 3
translation 4
Cellular Component
mitochondrial small ribosomal subunit 1
endoplasmic reticulum 1
cytoskeleton 1
microtubule 1
adherens junction 1
integral component of membrane 1
myosin complex 1
extrinsic component of membrane 1
dynein complex 1
keratin filament 1
postsynaptic membrane 1
proteasome core complex 2
intermediate filament 2
Arp2/3 protein complex 2
actin cytoskeleton 2
membrane 2
ribosome 4
cytoplasm 5
intracellular 7
Molecular Function
kinase activity 1
oxidoreductase activity 1
hydrolase activity 1
heme binding 1
acetylcholine–gated cation–selective channel activity 1
manganese ion binding 1
unfolded protein binding 1
NAD+ binding 1
threonine–type endopeptidase activity 2
structural molecule activity 2
GTP binding 2
protein domain specific binding 2
ribosome binding 2
RNA binding 3
actin filament binding 3
nucleic acid binding 4
structural constituent of ribosome 4
ATP binding 4
calcium ion binding 6
protein binding 13
0
5
10
15
Number of Protein
